# Status and Challenges of Cathode Materials for Room‐Temperature Sodium–Sulfur Batteries

**DOI:** 10.1002/smsc.202100059

**Published:** 2021-09-24

**Authors:** Ying Wu, Liang Wu, Shufan Wu, Yu Yao, Yuezhan Feng, Yan Yu

**Affiliations:** ^1^ Hefei National Laboratory for Physical Sciences at the Microscale Department of Materials Science and Engineering CAS Key Laboratory of Materials for Energy Conversion University of Science and Technology of China Hefei Anhui 230026 China; ^2^ Key Laboratory of Materials Processing and Mold (Zhengzhou University) Ministry of Education Zhengzhou University Zhengzhou 450002 China; ^3^ National Synchrotron Radiation Laboratory Hefei Anhui 230026 China

**Keywords:** adsorption–catalysis, room-temperature sodium–sulfur batteries

## Abstract

Room‐temperature sodium–sulfur (RT Na–S) batteries have become the most potential large‐scale energy storage systems due to the high theoretical energy density and low cost. However, the severe shuttle effect and the sluggish redox kinetics arising from the sulfur cathode cause enormous challenges for the development of RT Na–S batteries. This review systematically sheds light on the rational design strategies of integrating porous carbon matrix with “adsorption–catalysis” agents, including transition‐metal single‐atom, transition‐metal nanoclusters, transition‐metal compounds, or heterostructures. Moreover, the multistep reaction mechanism accompanied with the evolution process of various sodium polysulfides during the redox process is systematically summarized on the basis of electrochemical technique analysis and ex situ/in situ characterization. Finally, the future perspectives and potential research directions are outlined to provide a guideline for the continuous development of RT Na–S batteries.

## Graphical abstract

This review sheds light on the rational design strategies of integrating porous carbon matrix with “adsorption–catalysis” agents for room‐temperature sodium‐sulfur batteries (RT Na–S). The multi‐step reaction mechanism during the redox process is systematically summarized. Future perspectives and research directions are outlined to provide a guideline for the development of RT Na–S batteries.

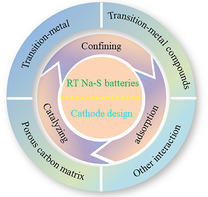

## 1 Introduction

The rapid development of electric vehicles, smart grids, clean energy, and other fields has promoted the progress of advanced energy storage technology (e.g., secondary batteries).^[^ 1, 2, 3, 4 ^]^ Among them, rechargeable lithium‐ion batteries (LIBs) have successfully powered these equipments.^[^ 2, 5, 6, 7 ^]^ While the energy density of commercial LIBs has reached their theoretical ceilings.^[^ 5, 8 ^]^ Therefore, the exploitation of lithium–sulfur (Li–S) batteries,^[^ ^9^ ^]^ lithium–air batteries^[^ ^10^ ^]^ as well as room‐temperature sodium–sulfur (RT Na–S) batteries^[^ ^11^ ^]^ with ultrahigh theoretical energy density holds great promise in powering the large‐scale stationary energy storage system. In light of the scarce lithium resources and unevenly distribution around the world, it is keen to develop RT Na–S batteries with the sulfur cathode and sodium anode, holding the advantages of abundant resources and low cost.^[^ ^12^ ^]^ Moreover, based on the two‐electron conversion reaction mechanism, RT Na–S batteries present ultrahigh theoretical capacity of 1675 mAh g^−1^ and energy density of 1274 Wh kg^−1^.^[^ 13, 14, 15 ^]^ However, the actual energy density and specific capacity of RT Na–S batteries are far from the theoretical values. In addition, the long cycling performance is still unsatisfying. In the final analysis, similar with the Li–S batteries, the sluggish reaction kinetics of the nonconductive sulfur and the discharge products of Na_2_S_2_ and Na_2_S, as well as severe “shuttle effect” at the sulfur cathode become the enormous challenges to hinder the practical application of RT Na–S batteries.^[^ 16, 17, 18 ^]^ As the inferior performance of RT Na–S batteries mainly comes from sulfur cathode, it is urgent to develop effective modification strategies to improve it.

In the past few decades, the modification strategies for the sulfur cathode of RT Na–S batteries are mainly inspired from the achievements in the Li–S batteries.^[^ 19, 20, 21, 22, 23, 24, 25 ^]^ However, compared with Li–S batteries, the RT Na–S batteries would face much greater obstacle due to the relatively larger Na^+^, causing rather sluggish redox kinetics, huge volume change, and fast capacity decay.^[^ 16, 26, 27, 28, 29, 30 ^]^ To solve these issues in the sulfur cathode, research on its modification mainly focuses on the following aspect.^[^ ^31^ ^]^ At the very beginning, the encapsulation of sulfur molecular into the pores of various carbon host enables certain improvement of electrochemical performance, which benefits from the physical confinement effect of the porous carbon host, the enhanced electronic conductivity, as well as the alleviation of volume change and shuttle effect.^[^ 32, 33, 34 ^]^ Unfortunately, the interaction between the carbonaceous matrix and the soluble intermediates of sodium polysulfides (NaPSs) by van der Waals force is too weak to realize effective suppression.^[^ ^35^ ^]^ Afterward, to enhance the interaction force, the introduction of chemical interaction has drawn great research interest.^[^ 15, 36 ^]^ The design of polar host, e.g., modification the porous carbon matrix via heteroatoms doping or the introduction of functional groups, can enhance the adsorption energy with NaPSs. Another approach is the integration of polar substance such as metal or metal compounds into carbon matrix, which provide abundant adsorption active sites.^[^ 37, 38 ^]^ However, based on the aforementioned strategies, the “shuttle effect” of NaPSs could be only suppressed to some extent. In other words, the dissolution and diffusion of NaPSs cannot be eliminated fundamentally based on the idea of blocking.

Gaining deep insight into the multistep conversion mechanism of the intermediate NaPSs, the acceleration of the reduction and oxidation kinetics of polysulfides is the key issue.^[^ 27, 39 ^]^ Inspired form the concept of electrocatalysis,^[^ 40, 41 ^]^ various electrocatalytically active materials haven been successfully introduced into porous carbon hosts to synergistically alleviate the shuttle effect and enhance the conversion reaction kinetics.^[^ ^42^ ^]^ Thus, construction of sulfur hosts with confinement–adsorption–catalysis effects could effectively improve the electronic conductivity, the ion diffusion kinetics, and the rapid conversion of solvable NaPSs to the final reduction products of Na_2_S_2_/Na_2_S, resulting in satisfying performance for the RT Na–S batteries. The electrocatalytically active materials mainly contain metal,^[^ ^15^ ^]^ metal carbides,^[^ ^43^ ^]^ metal nitrides,^[^ ^44^ ^]^ metal oxides,^[^ ^45^ ^]^ and metal sulfides.^[^ ^46^ ^]^ Although the enhanced electrochemical performance has been realized with the assistance of various electrocatalytically active materials, the adsorption–catalysis mechanism during the complex multistep reaction process still remains unclear. Generally, the ex situ characterization cannot accurately reflect the actual state of the battery due to the sensitivity of the intermediate reaction products to the external environment. Therefore, in situ characterization, such as in operando X‐ray diffraction (XRD), in situ UV–vis spectroscopy, in situ Raman and in situ X‐ray absorption near edge structure (XANES), are developed to real‐time monitor the catalysis–conversion mechanism during the discharge/change process.^[^ ^47^ ^]^

With these considerations, this Review focuses on recent advances on the sulfur cathode matrix for RT Na–S batteries. Meanwhile, we also provide a comprehensive and specialized overview on the design principles of effective sulfur cathodes and get insight into the adsorption–catalysis mechanism via advanced in situ/ex situ characterizations. In addition, the corresponding theoretical calculations are deeply analyzed to understand electrochemical reaction behaviors. Finally, challenges and opportunities for the development of RT Na–S batteries are put forward.

## 2 Electrochemistry and Challenges of RT Na–S Batteries

### 2.1 Principles of RT Na–S Batteries

The configuration of RT Na–S batteries are composed of sulfur‐based composites as the cathode and sodium metal as the anode. RT Na–S batteries possess a multistep reaction process with complicated intermediates.^[^ ^48^ ^]^ During the discharge process, the cyclic octatomic sulfur ring (S_8_) is first reduced and reacts with Na^+^ to form soluble long‐chain NaPSs (Na_2_S_*x*_, 4 ≤ *x* ≤ 6), followed by further transformation of long‐chain NaPSs to short‐chain NaPSs and finally to Na_2_S_2_/Na_2_S.^[^ ^31^ ^]^ Therefore, getting deep insight into the complex multistep reaction process is the key for achieving the high‐performance RT Na–S batteries.

### 2.2 Challenges in RT Na–S Batteries

As the complicated multistep reaction process, the obstacles of RT Na–S batteries mainly lie in as following.

#### 2.2.1 The Sluggish Conversion Reaction Kinetics

The insulation of sulfur and the discharge products of Na_2_S_2_/Na_2_S would cause poor sodium‐ion diffusion kinetics and low utilization of sulfur, resulting in low Coulombic efficiency.^[^ ^49^ ^]^ Moreover, the evolution of sulfur species during the redox reaction process is even more kinetically difficult in RT Na–S batteries than that of Li–S batteries due to the larger sodium‐ion radius.^[^ ^50^ ^]^ Worse still, the slower conversion reaction rate would bring the accumulation of soluble NaPSs to deepen shuttle effect.

#### 2.2.2 Severe Shuttle Effect

During the discharge process, the intermediate products of long‐chain NaPSs are soluble in electrolyte, passing through the separator and reaching the sodium metal anode, and finally reduced to the insulating short‐chain NaPSs, resulting in the continuous loss of sulfur active materials, which causes successive degradation of the battery capacity and Coulombic efficiency.^[^ 16, 26, 51 ^]^ At the same time, the deposition of the insulating short‐chain NaPSs on the sodium metal anode would cause the corrosion of sodium metal anode and the increase in electrode impedance.^[^ ^52^ ^]^ Moreover, the dissolution of long‐chain NaPSs would arouse serious self‐discharge phenomenon.^[^ ^32^ ^]^

#### 2.2.3 Huge Volume Variation

Due to the different densities of the NaPSs intermediates, it will lead to a large volume variation of the electrode, which poses great challenge to the stability of the electrode structure. In addition, the volume expansion could be even up to ≈170%.^[^ ^17^ ^]^

**Figure 1:**
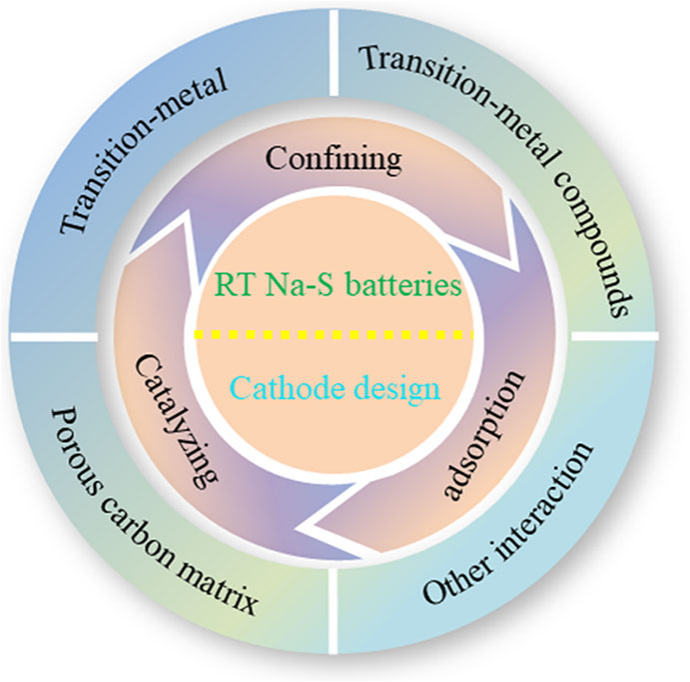
Schematic illustration of the modification strategies of sulfur cathode for RT Na–S batteries.

Considering the challenges in RT Na–S batteries toward the practical application, it is particularly urgent to develop effective modification strategies to overcome the aforementioned obstacles. The design principles of advanced sulfur cathode mainly focus on the synergistic effect of physical confinement, chemical adsorption, and catalytic conversion combined with the highly conductive porous carbon matrix (**Figure** **1**). Moreover, getting insight into the multistep mechanism of the RT Na–S electrochemical system is of great significance.

## 3 Porous Carbon Matrix for Sulfur Cathode with Confinement/Adsorption Effects

In view of the challenges in the sulfur cathode of RT Na–S batteries, various strategies focus on modifying the sulfur cathode have been developed to enhance the conversion kinetics and alleviate the shuttle effect. In recent years, porous carbon matrix applied as sulfur/polysulfides reservoir has made great process.^[^ 53, 54, 55 ^]^ The highly conductive carbon matrix with abundant porous structure or polar species plays key role in alleviating the volumetric swelling as well as physically confining the dissoluble intermediates of NaPSs to avoid shuttle effect. In addition, the construction of novel carbonaceous host with high surface area could endow the improved electronic conductivity with fast electron/charge transfer and accommodate the sulfur active material with high mass loading.^[^ ^24^ ^]^ Actually, the existence form of sulfur molecules depends on the pore size of the carbon host, which shows great influence on the intermediate process of the multistep reactions.

### 3.1 Microporous Carbon Matrix

**Figure 2:**
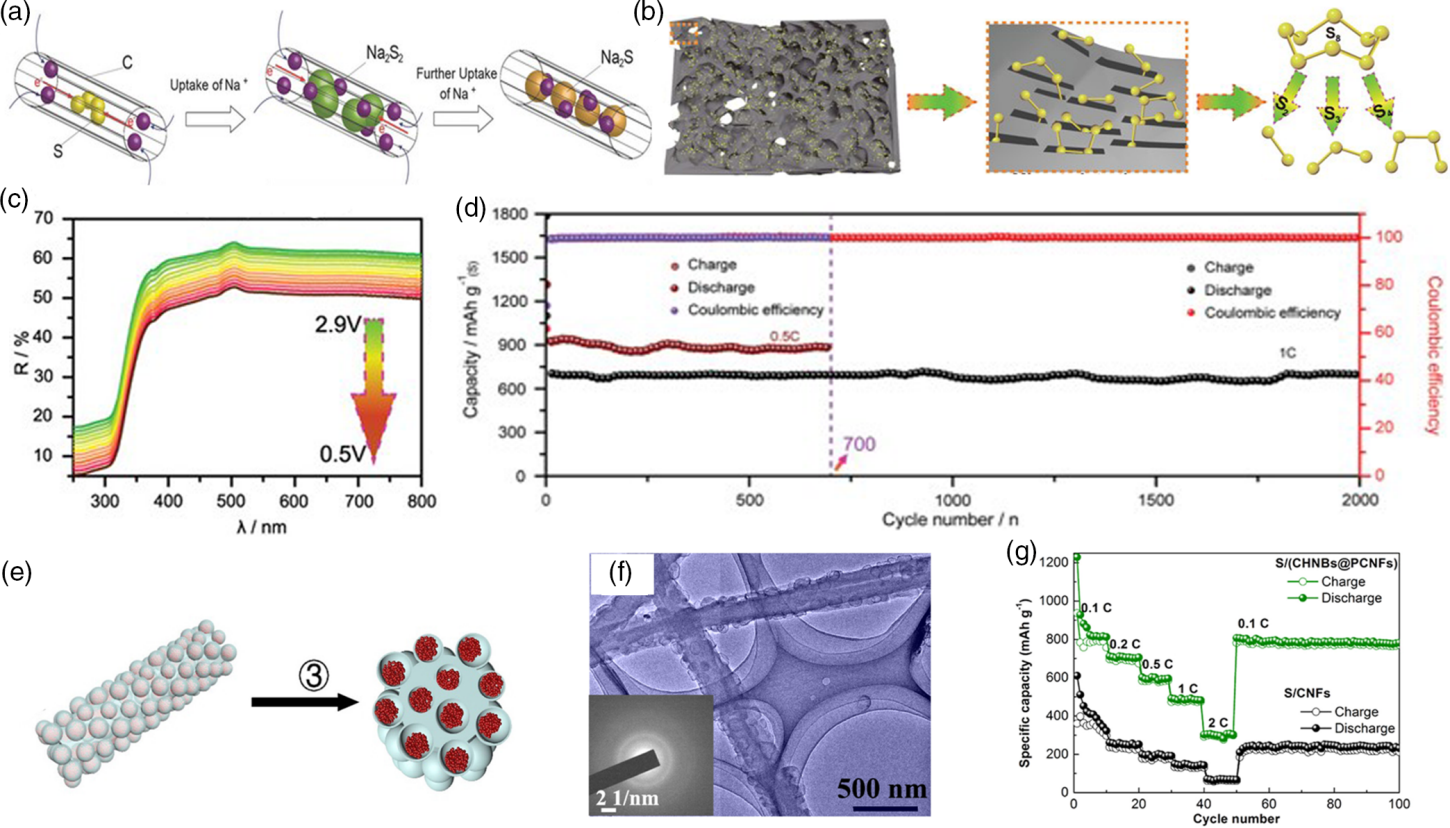
a) Schematic illustration and electrochemical reactions of sulfur with Na^+^ during discharge process; Reproduced with permission.^[^ ^57^ ^]^ Copyright 2013, Wiley‐VCH. b) The confinement of small sulfur molecules (S_2–4_) in the slit ultramicropore carbon matrix; c) In situ UV–vis spectra at 0.1 C during discharge process; d) Long cycle performances at 0.5 and 1 C; b–d) Reproduced under the terms of the CC‐BY 4.0 license.^[^ ^53^ ^]^ Copyright 2020, The Authors, published by Wiley‐VCH. e) Schematic illustration of the synthesis process of C/S composite and f) the corresponding TEM image; g) Rate performance at different current densities for the as‐synthesized C/S cathode. e–g) Reproduced with permission.^[^ ^58^ ^]^ Copyright 2018, Elsevier.

The micropores (< 2 nm) are regarded as ideal container to store small sulfur, thus preventing the formation of soluble long‐chain NaPSs and enabling stable cycling performance. Spherical aberration imaging technique indicates that metastable small sulfur molecules can be confined within micropores with diameter of 0.5 nm.^[^ ^56^ ^]^ For example, early in 2014, Wan's and coworkers^[^ ^57^ ^]^ reported the successful encapsulation of S_2–4_ sulfur within the conductive microporous carbon matrix with a core/porous‐sheath structure (S/(CNT@MPC). **Figure** **2**a shows the schematic illustration and electrochemical reactions of sulfur with Na^+^ during discharge process. The corresponding theoretical calculations as well as the X‐ray photoelectron spectroscopy (XPS) and transmission electron microscopy (TEM) analysis confirm the feasible reduction of small S_2–4_ into Na_2_S_2_ and Na_2_S, indicating no soluble long‐chain NaPSs formation and shuttle effect during cycling. Moreover, benefiting from the space confinement effect of micropores, the reduction product of Na_2_S cannot convert to S_8_ molecules during the oxidation process, but the formation of small S_2–4_ molecules. Overall, the high electrochemical activity of small S_2–4_ molecules as well as the geometry confinement effect of the well‐designed microporous carbon host endow rather stable cycling ability and outstanding rate performance. However, the insulated intermediate reaction product of Na_2_S_2_ still exists, which slows down the reaction kinetics. Therefore, the design of unique cathode matrix that occurred one‐step reaction with Na_2_S as the only sodiation product as well as avoiding the formation of soluble NaPSs shows great promise. In the latest study, Xia and coworkers^[^ ^53^ ^]^ proposed the one‐step mechanism of the RT Na–S batteries with the slit ultramicropore carbon (derived from the coffee residual) as host to confine small sulfur molecules (S_2–4_) through a traditional melting‐diffusion method (Figure 2b). Density functional theory (DFT) calculations indicated that small sulfur molecules (S_2–4_) exist in the slit ultramicropores with the diameter of 0.5 nm. The electrochemical measurement as well as ex situ or in situ characterization, such as the in situ UV–vis spectroscopy in Figure 2c, further confirm the one‐step reaction mechanism with the only reduction product of Na_2_S. As shown in Figure 2d, this unique structure presents ultrahigh specific capacity (1110 mAh g^−1^ remained after 400 cycles at 0.1 C) and superior cycling stability (small capacity decay even up to 2000 cycles). However, when the small sulfur molecule is encapsulated into microporous carbon matrix, only a limited sulfur content (generally lower than 40%) could be achieved, resulting in low energy density of the full battery.

### 3.2 Hierarchical Porous Carbon Matrix

Compared with microporous carbon, the encapsulation of sulfur into mesoporous carbon matrix could enhance the sulfur loading and the infiltration of electrolyte, whereas the dissolution of long‐chain sodium polysulfides should be considered.^[^ ^27^ ^]^ Therefore, the design of hierarchical porous carbon matrix with the synergistic exploitation of different types of pores shows great promise. For example, Yu and coworkers^[^ ^58^ ^]^ designed the hierarchical porous carbon nanofibers decorated with hollow carbon nanobubbles as sulfur host via the bubbling‐assisted electrospinning strategy and subsequent the heat‐treatment process (Figure 2e). Benefiting from the bubble agent of Li_3_N, the hierarchical pores including micropores, mesopores, and macropores were obtained (Figure 2f). In addition, the 3D‐interconnected network structure of the carbon matrix endows a high electronic conductivity. Therefore, as shown in Figure 2g, the as‐synthesized C/S composite cathode exhibits excellent high‐rate capability (up to 304 mAh g^−1^ even at 2 C). In addition, Liu and coworkers^[^ ^59^ ^]^ reported a passion fruit‐like double‐shell porous carbon host (PCM) with hierarchical pores for the RT Na–S batteries through the sacrificial template method. The inner hollow mesopores guarantee for high sulfur loading. At the meantime, the abundant microporous in the out shell provide a barrier to prevent the dissolution of long‐chain NaPSs. Ascribe to the hierarchical porous carbon matrix, the PCM‐S cathode displays good rate performance.

As mentioned earlier, the microporous carbon matrix as well as the synergistic effects of hierarchical structure can restrain the dissolution of soluble NaPSs via physical confinement, providing a stable long cycling life span.

### 3.3 Polar Functional Groups Modified Carbon Matrix

**Figure 3:**
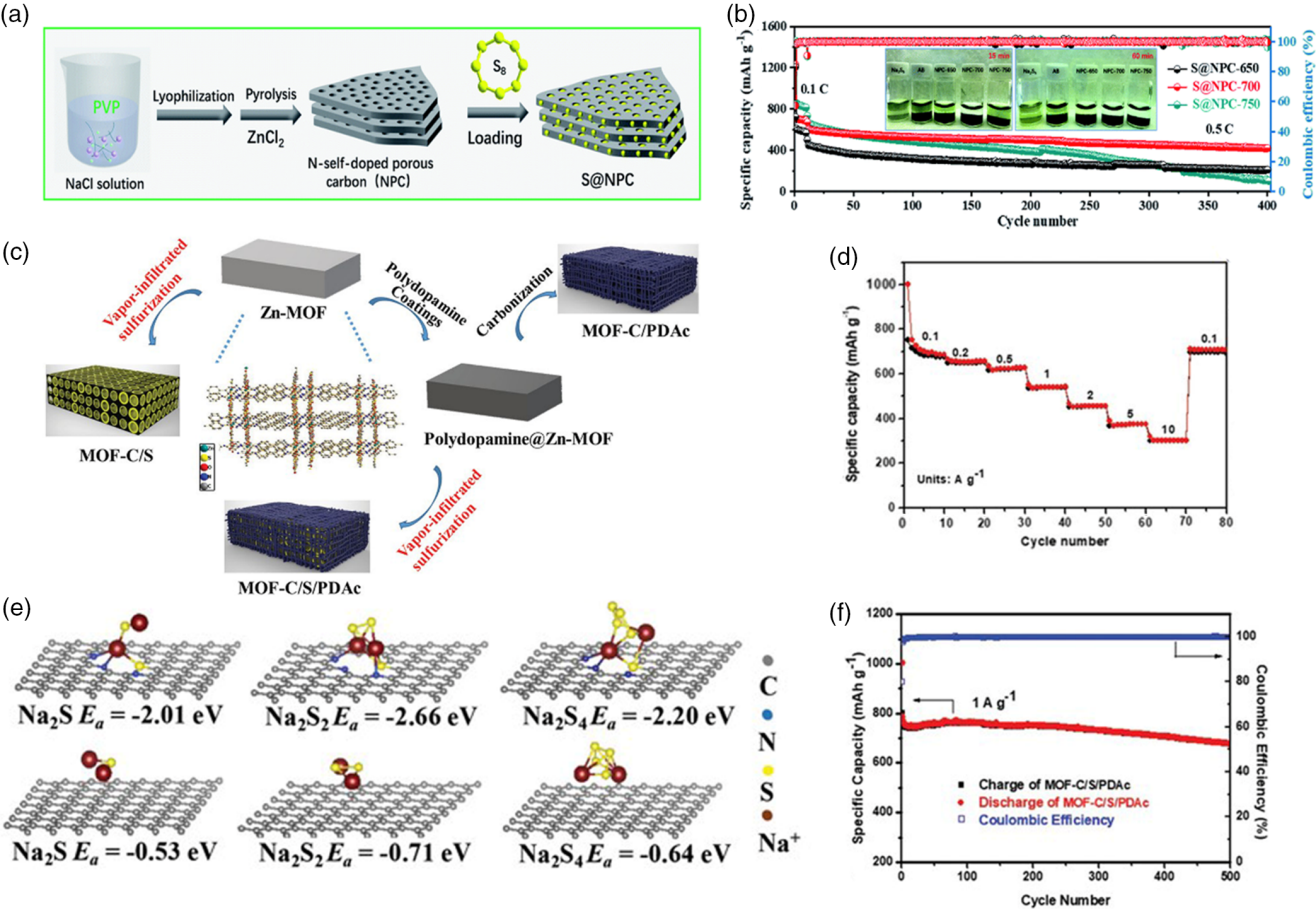
a) Schematic illustration of the synthesis process of the hierarchical porous carbon sheets host confined with sulfur; b) the corresponding long cycling performance at 0.5 C; a,b) Reproduced with permission.^[^ ^33^ ^]^ Copyright 2020, Royal Society of Chemistry. c) Fabrication process and schematics of the N, S codoped microporous carbon matrix confined with sulfur; d,f) The corresponding rate capability and long cycling performance at 1 A g^−1^; e) adsorption energies for NaPSs species on the N, S codoped carbon and pristine carbon species. c–f) Reproduced with permission.^[^ ^60^ ^]^ Copyright 2020, Wiley‐VCH.

Although the physical confinement effect of the porous carbon matrix can optimize the cycle stability of the RT Na–S batteries, the interaction based on van der Waals force is not sufficient to suppress the dissolution of polysulfides. Therefore, the introduction of chemical interaction to effectively immobilize NaPSs is very critical. Modification the porous carbon matrix via heteroatoms doping or the introduction of functional groups could enhance the adsorption of soluble NaPSs. For example, Liu and coworkers^[^ ^33^ ^]^ successfully developed hierarchically nitrogen‐doped porous carbon sheets (using polyvinyl pyrrolidone [PVP] as carbon and nitrogen source) as sulfur host for RT Na–S batteries (**Figure** **3**a). The heteroatoms doping of nitrogen could effectively enhance the electronic conductivity and further improve the charge transfer kinetics. Benefiting from the confinement effect of the micropores to immobilize sulfur species and the chemical adsorption effect of nitrogen doping to alleviate the dissolution of NaPSs, high reversible capacity of 418.9 mAh g^−1^ at 0.5 C (Figure 3b) and excellent rate performance were achieved. Meanwhile, Xiao et al.^[^ ^60^ ^]^ designed and fabricated the N, S codoped microporous carbon matrix as sulfur host through the carbonization of the 3D Zn‐MOF and polydopamine coating layer. The synthetic procedure based on the vapor‐infiltration strategy is shown in Figure 3c. The XPS and solid‐state NMR tests revealed that sulfur exists in the form of the covalent bonding (C—S_*x*_—C), which could enhance the chemical interaction between sulfur species and polar carbon matrix to prevent the dissolution of NaPSs. As shown in Figure 3e, the DFT results confirmed that the S, N‐doped carbon matrix shows more negative adsorption energies for various Na_2_S_*x*_ species compared with the nondoped one. Superior electrochemical performance with high reversible capacity of 467 mAh g^−1^ at 0.1 A g^−1^, stable long cycling life over 1000 cycles at 1 A g^−1^ and excellent rate performance could be achieved (Figure 3d,f).

## 4 Polar Materials Modified Carbon Matrix with Adsorption–Catalysis Effects

Except for modifying carbonaceous host with polar functional groups, the integration of polar component into nonpolar porous carbon hosts is confirmed to be a more effective strategy, in which the polar active material shows strong adsorption energy with NaPSs species.^[^ 22, 27 ^]^ The rate‐determining step in RT Na–S batteries is the conversion of polysulfide to sodium sulfide during the reduction process and the recovery of sulfur during the subsequent oxidation process. Advanced strategies to improve the kinetics of NaPSs conversion reaction during the charge/discharge process are thus crucial to avoid the accumulation of soluble NaPSs in the cathode and alleviate severe shuttle effect. Furthermore, most of the polar materials also possess the ability to catalyze the fast conversion of long‐chain NaPSs to short‐chain NaPSs with enhanced reaction kinetics, which indicates the future research direction of RT Na–S batteries by innovative bridging battery and electrocatalyst fields.^[^ 29, 61 ^]^ The adsorption interaction and catalysis effects synergistically strengthen the immobilization of soluble long‐chain NaPSs and subsequently accelerate the conversion between polysulfides during the reduction/oxidation processes. Therefore, the design of ideal sulfur host with highly conductive network, abundant hierarchical pores as well as polar centers being capable of adsorption–catalysis effects is highly attractive. In this section, recent research progress on electrocatalysis for RT Na–S batteries would be analyzed.

### 4.1 Transition‐Metal Catalysts

#### 4.1.1 Nanosized Transition‐Metal Catalysts

**Figure 4:**
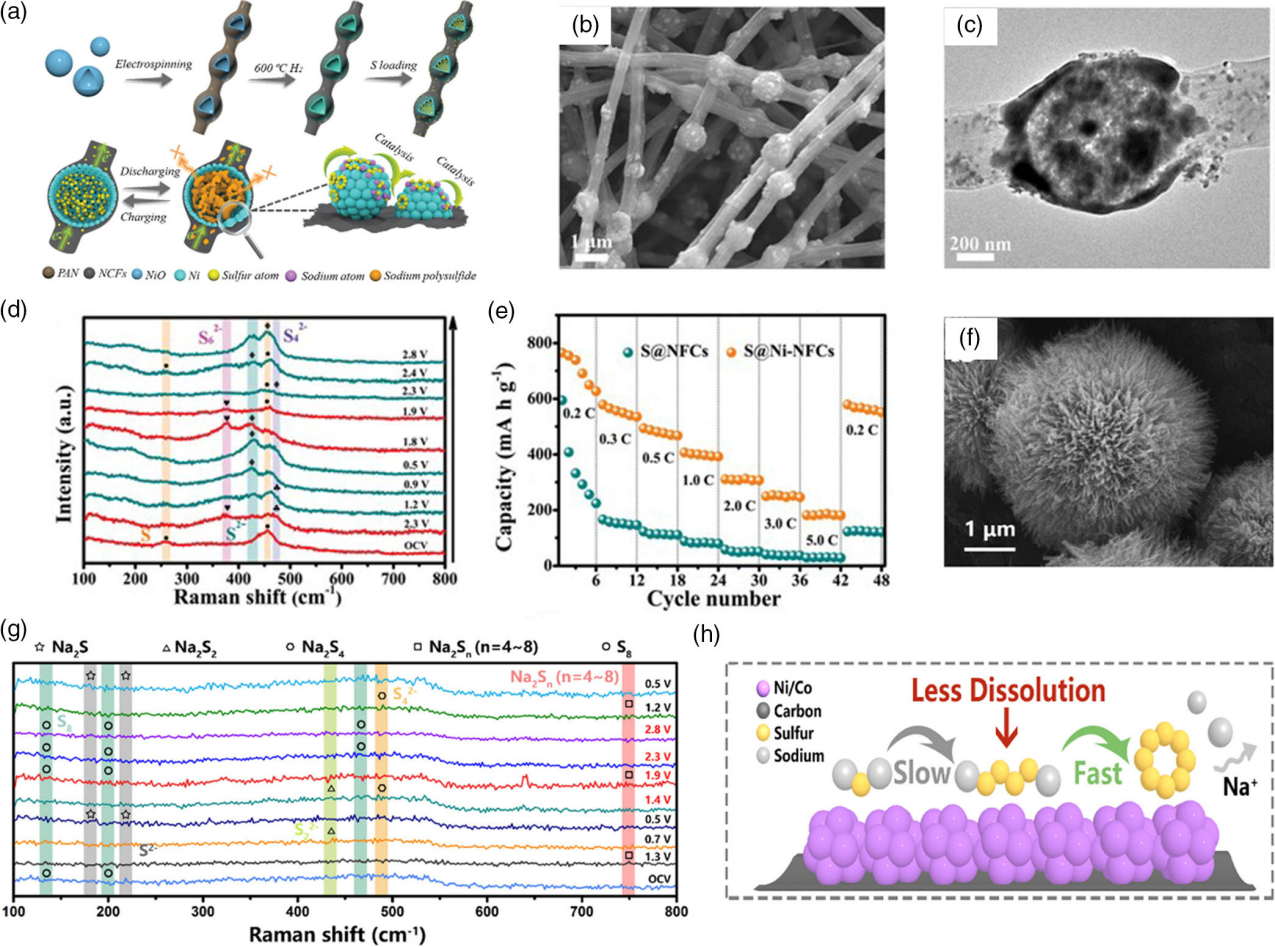
a) Schematic illustration for the synthesis of the S@Ni‐NCFs composite and the advantages for RT Na–S batteries; b) SEM and c)TEM images of the S@Ni‐NCFs composite; d) In situ Raman and e) rate capability of the S@Ni‐NCFs electrode; a–e) Reproduced under the terms of the CC‐BY 4.0 license.^[^ ^62^ ^]^ Copyright 2019, The Authors, published by Wiley‐VCH. f) SEM image of the S@Ni/Co‐C‐12 composite; g) In situ Raman analysis of the S@Ni/Co‐C electrode; h) the advantages of the Ni/Co bimetal during the conversion process. f–h) Reproduced with permission.^[^ ^63^ ^]^ Copyright 2021, American Chemical Society.

Xu and coworkers^[^ ^62^ ^]^ designed and fabricated the unique 1D nitrogen‐doped carbon nanofibers confined with Ni hollow spheres as a free‐standing sulfur host (S@Ni‐NCFs) for RT Na–S batteries. As shown in **Figure** **4**a–c, the Ni hollow nanospheres composed of Ni nanoparticles are well wrapped in the 3D‐intertwined carbon nanofibers, which endow the sulfur matrix with smooth electron ﬂow along different directions and alleviate the volume variation during the conversion process of polysulfides. As confirmed by the cyclic voltammetry (CV) of symmetric cell in Na_2_S_6_ electrolyte and in situ Raman measurements (Figure 4d), the existence of metallic Ni sites along with the partial‐formed polar Ni—S bonds could effectively strengthen the interfacial affinity with polysulfides via chemisorption and accelerate the conversion kinetics of sodium polysulfides. Benefiting from the structure merits along with the adsorption–catalysis effects of Ni nanoparticles, S@Ni‐NCFs cathode demonstrates excellent rate performance (e.g., showing high specific capacity of 249.8 mAh g^−1^ at 3 C, Figure 4e) and long cycling stability with the maintenance of 233 mAh g^−1^ after 270 cycles at 1 C. Meanwhile, Li and coworkers^[^ ^63^ ^]^ reported that by adjusting the composition of Ni/Co bimetallic catalyst (Figure 4f shows the SEM image of the Ni/Co precursor), the conversion kinetics of a two‐step mechanism from Na_2_S to NaPSs and to sulfur could be tailored, leading to rapid conversion kinetics and weak shuttle effect. By turning the Ni/Co molar ratio to 1:2, the S@Ni/Co‐C‐12 cathode exhibits the highest reversible capacity as well as the best long‐term cycle performance with 414.4 mAh g^−1^ after 650 cycles. As shown in Figure 4g, in situ Raman results reveal the reversible multistep reaction mechanism with the formation of reductive products of Na_2_S_*n*_ (*n* = 4–8), Na_2_S_2_ and final Na_2_S. CV profiles disclose the fast conversion process from long‐chain NaPSs to the final Na_2_S product. In addition, galvanostatic intermittent titration (GITT) test reveals fast Na^+^ transfer kinetics within the well‐designed S@Ni/Co‐C‐12 cathode. Therefore, as shown in Figure 4h, the well‐designed highly conductive porous carbon matrix along with suitable adsorption–catalysis effects plays key role for such high‐performance sulfur cathode.

#### 4.1.2 Transition‐Metal Single‐Atom or Metal Nanocluster Catalysts

**Figure 5:**
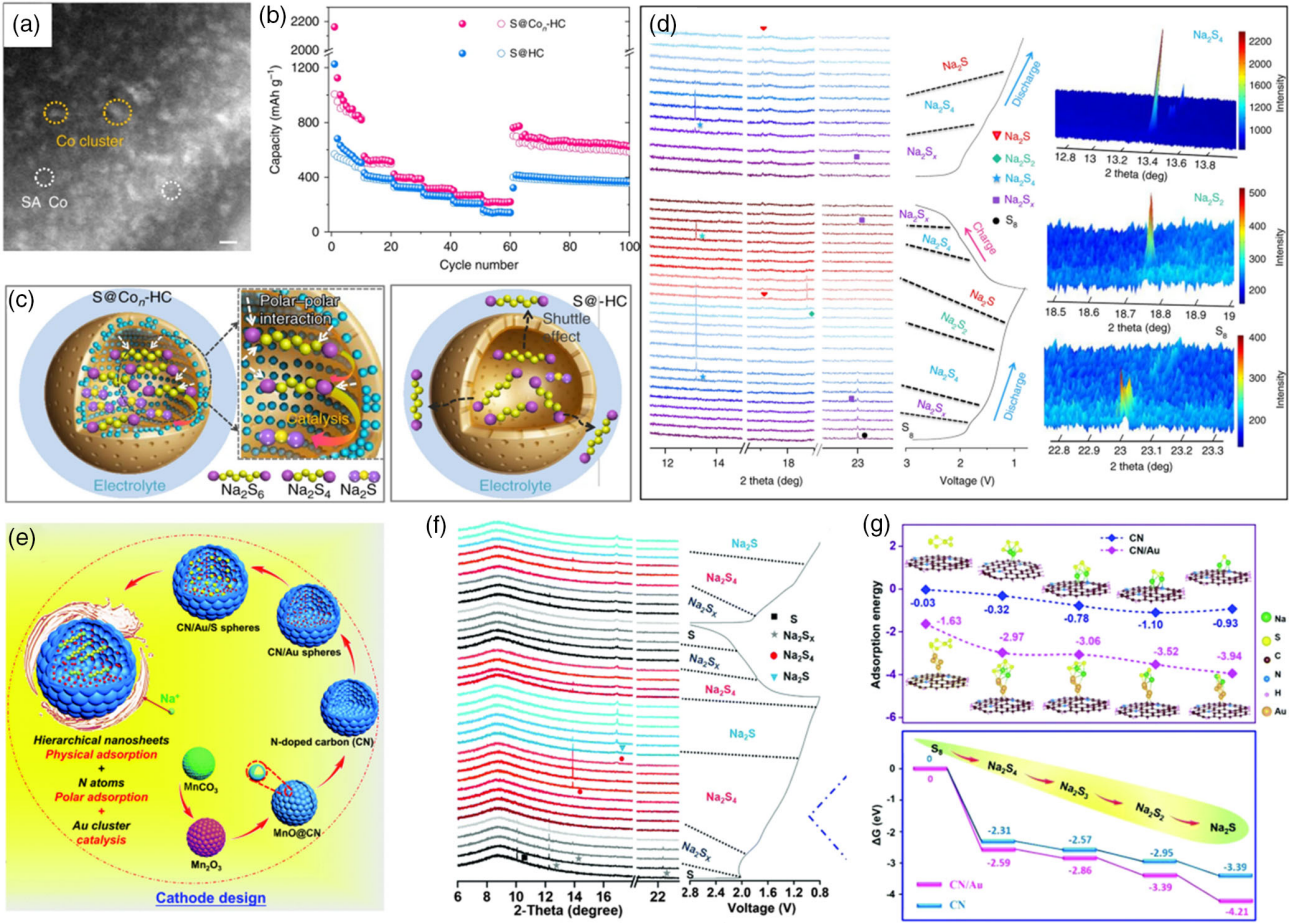
a) High‐angle annular dark‐field scanning transmission electron microscopy (HADDF‐STEM) image of the S@Co_n_‐HC composite; b) Rate performance for the S@Co_n_‐HC cathode; c) Schematic illustrations of the reaction mechanism of the S@Co_n_‐HC cathode; d) In situ synchrotron XRD patterns of the S@Co_n_‐HC cathode; a–d) Reproduced under the terms of the CC‐BY 4.0 license.^[^ ^37^ ^]^ Copyright 2018, The Authors, published by Springer Nature. e) Schematic of the advantages of the CN/Au/S cathode in RT Na–S batteries; f) in situ synchrotron XRD patterns of the CN/Au/S cathode during charge/discharge process; g) the adsorption energies and the Gibbs free energies of NaPSs on the nitrogen‐doped carbon surface and Au‐decorated nitrogen‐doped carbon. e–g) Reproduced with permission.^[^ ^49^ ^]^ Copyright 2020, Royal Society of Chemistry.

Transition‐metal single‐atom catalysts (SACs) and metal clusters well‐dispersed on the porous carbon matrix with maximum atom efficiency and well‐defined active sites already became an effective strategy to realize efficient catalysis.^[^ ^64^ ^]^ Inspired by this, SACs/metal clusters hold significant promise to realize the alleviation of shuttle effect and fast conversion reaction kinetics in RT Na–S batteries. For example, as shown in **Figure** **5**a–c, Zhang et al.^[^ ^37^ ^]^ prepared the microporous hollow carbon nanospheres with well‐dispersed atomic cobalt as the host of sulfur cathode (S@Co_*n*_‐HC) to immobilize the NaPSs via the formation of Co—S bond and electrocatalytically reduce the long‐chain NaPSs to Na_2_S. XPS measurement detects the coexistence of Co—S bond and Co^0^, leading to the enhanced electronic conductivity and interaction with sulfur species. To make deep understanding on the mechanism of unique sulfur cathode, CV, in situ Raman spectroscopy as well as in situ synchrotron XRD (Figure 5d) were conducted and revealed the two‐step reaction mechanism with the reduction of solid sulfur to soluble long‐chain NaPSs and then to short‐chain Na_2_S_*y*_ (1 < *y* ≤ 3). In particular, the in situ synchrotron XRD pattern of the second discharge process in Figure 5d shows the weak peak intensity of Na_2_S_4_ and the absence of Na_2_S_2_ intermediate products, suggesting that atomic Co could enhance the conversion kinetics from Na_2_S_4_ to Na_2_S. Ab initio molecular dynamics (AIMD) simulations were conducted to reveal the adsorption process of polysulfides species on the Co_6_ clusters and pure carbon. The theoretical result indicates the strong adsorption energy of polysulfides species on Co_6_ clusters and the conversion from Na_4_S_2_ to Na_2_S is kinetically fast. When used in RT Na–S batteries, high reversible capacity (e.g., 1081 mAh g^−1^ at 100 mA g^−1^), stable long cycling life (maintaining even 508 mAh g^−1^ after 600 cycles) and excellent rate performance (e.g., showing high reversible capacity of 220.3 mAh g^−1^ even at 5 A g^−1^) could be achieved. In their later study,^[^ ^65^ ^]^ the electrocatalysis effects of Fe, Cu, and Ni nanoclusters well‐dispersed on hollow carbon spheres as the host of sulfur cathode were further evaluated, demonstrating similar adsorption effects as well as electrocatalysis mechanism with atomic Co. The AIMD simulations reveal that Fe nanoclusters possess the most suitable reactivity with efficient confinement effect and catalytical activity for the transformation of long‐chain NaPSs to short‐chain NaPSs. Apart from the Fe, Co, Ni, and Cu nanoclusters, gold (Au) nanodots were also studied to evaluate the adsorption–catalysis effects in RT Na–S batteries.^[^ ^49^ ^]^ The hierarchical porous N‐doped carbon microspheres composed of crosslinked 2D nanosheets and well‐dispersed gold nanodots of around 1.8 nm (CN/Au/S) were successfully designed and fabricated as sulfur host (Figure 5e). The CN/Au matrix with high surface area and pore volume could serve as sulfur reservoir to physically and chemically alleviate the dissolution of long‐chain NaPSs. In situ synchrotron XRD (Figure 5f) and ex situ XRD tests confirm the catalysis function of gold nanodots on fully catalytic conversion of Na_2_S_4_ to Na_2_S product during the discharge process as well as complete recovery to sulfur during the subsequent charge process. Furthermore, as shown in Figure 5g, DFT calculations demonstrate that the CN/Au matrix shows strong adsorption capability with sulfur species through polar–polar interaction, which could effectively prevent the shuttling of NaPSs. In addition, the lower Gibbs free energy indicates the fast conversion kinetics due to the electrocatalysis effect of Au nanodots. When applied as cathode for RT Na–S batteries, the CN/Au/S cathode presents much higher specific capacity and stable long‐cycling life (430 mAh g^−1^ remained after 1000 cycles at 2 A g^−1^) as well as excellent rate capability (even up to 297 mAh g^−1^ at 10 A g^−1^). In addition, Lai et al.^[^ ^66^ ^]^ further reported the general synthesis of various SACs for RT Na–S batteries and studied the adsorption and catalytic ability through theoretical calculations and experiments. Similarly, the Fe@NC@S cathode shows the best adsorption and catalytic ability among the state‐of‐the‐art electrochemical performance as reported for RT Na–S batteries.

### 4.2 Catalysts of Transition‐Metal Compounds

In recent years, various polar transition‐metal compounds with strong adsorption capability and catalysis activity to accelerate the reaction kinetics have been attempted in Li–S batteries. Inspired by these, the application of transition‐metal compounds in RT Na–S batteries^[^ ^67^ ^]^ was also conducted.

#### 4.2.1 Transition‐Metal Oxides

**Figure 6:**
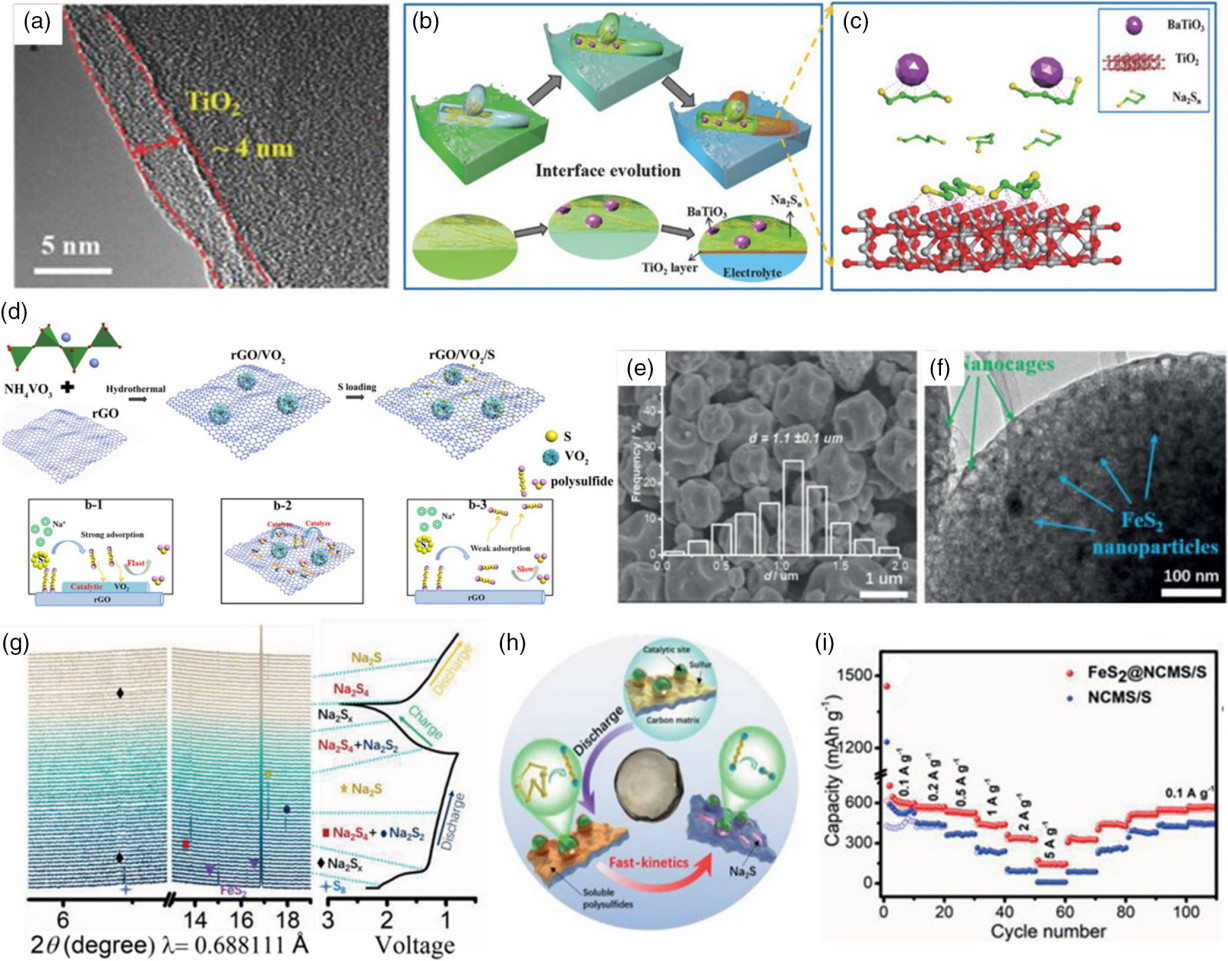
a) TEM images of the CSB@TiO_2_ nanofibers with TiO_2_ coating layer; b) the structural advantages of the BaTiO_3_ additive; and c) TiO_2_ layer deposition of the CSB@TiO_2_ cathode for RT Na–S batteries; a–c) Reproduced with permission.^[^ ^68^ ^]^ Copyright 2018, Wiley‐VCH. d) Schematic of the preparation and advantages of the rGO/VO_2_/S composite; Reproduced with permission.^[^ ^45^ ^]^ Copyright 2019, Elsevier. e) SEM and f) TEM images of the FeS_2_@NCMS/S composite; g) In situ synchrotron XRD patterns of the FeS_2_@NCMS/S electrode with the corresponding galvanostatic charge/discharge curves at the current density of 200 mA g^−1^; h) schematic illustrations of the sulfur conversion process and the electrocatalytic mechanism of the FeS_2_@NCMS/S cathode; i) rate performance of the FeS_2_@NCMS/S cathode. e–i) Reproduced with permission.^[^ ^70^ ^]^ Copyright 2020, Wiley‐VCH.

Transition‐metal oxides composed of polar metal—oxygen bonds could effectively absorb NaPSs and accelerate the conversion process. Early in 2018, Zhang and coworkers^[^ ^68^ ^]^ prepared the freestanding C/BaTiO_3_ nanofibers as the sulfur host via electrospinning. As shown in **Figure** **6**a, after the infiltration of sulfur via the sulfur melt‐diffusion process, a thin TiO_2_ layer with 4 nm thickness was coated on the C/S/BaTiO_3_ nanofibers via the atomic layer deposition (ALD) method (CSB@TiO_2_), and then used it as cathode for RT Na–S batteries. As shown in Figure 6b,c, the spontaneously polarized BaTiO_3_ nanoparticles could absorb the polar sodium polysulfdes. In addition, the thin TiO_2_ layer provides a smooth Na^+^ transport interface, displaying the reduced interfacial resistance and high adsorption capability toward soluble NaPSs. Benefiting from these synergistic effects, much stable long cycling life was achieved. What's more, in recent study, Xu and coworkers^[^ ^45^ ^]^ constructed a 3D metal oxide‐decorated carbon matrix (i.e., flower‐like VO_2_ microspheres in situ grown on the graphene oxide sheets [rGO/VO_2_/S]) for sulfur cathode (Figure 6d). CV and electrochemical impedance spectroscopy (EIS) measurements indicate the catalysis effect of VO_2_ with enhanced redox reaction kinetics. Up to now, the research on the catalysts of metal oxides for RT Na–S batteries is still rare without deep understanding on the mechanism. In addition, the relatively low electrical conductivity of metal oxides obstructs the charge transfer rate, leading to the restrained catalytic activity.

#### 4.2.2 Transition‐Metal Sulfides

**Figure 7:**
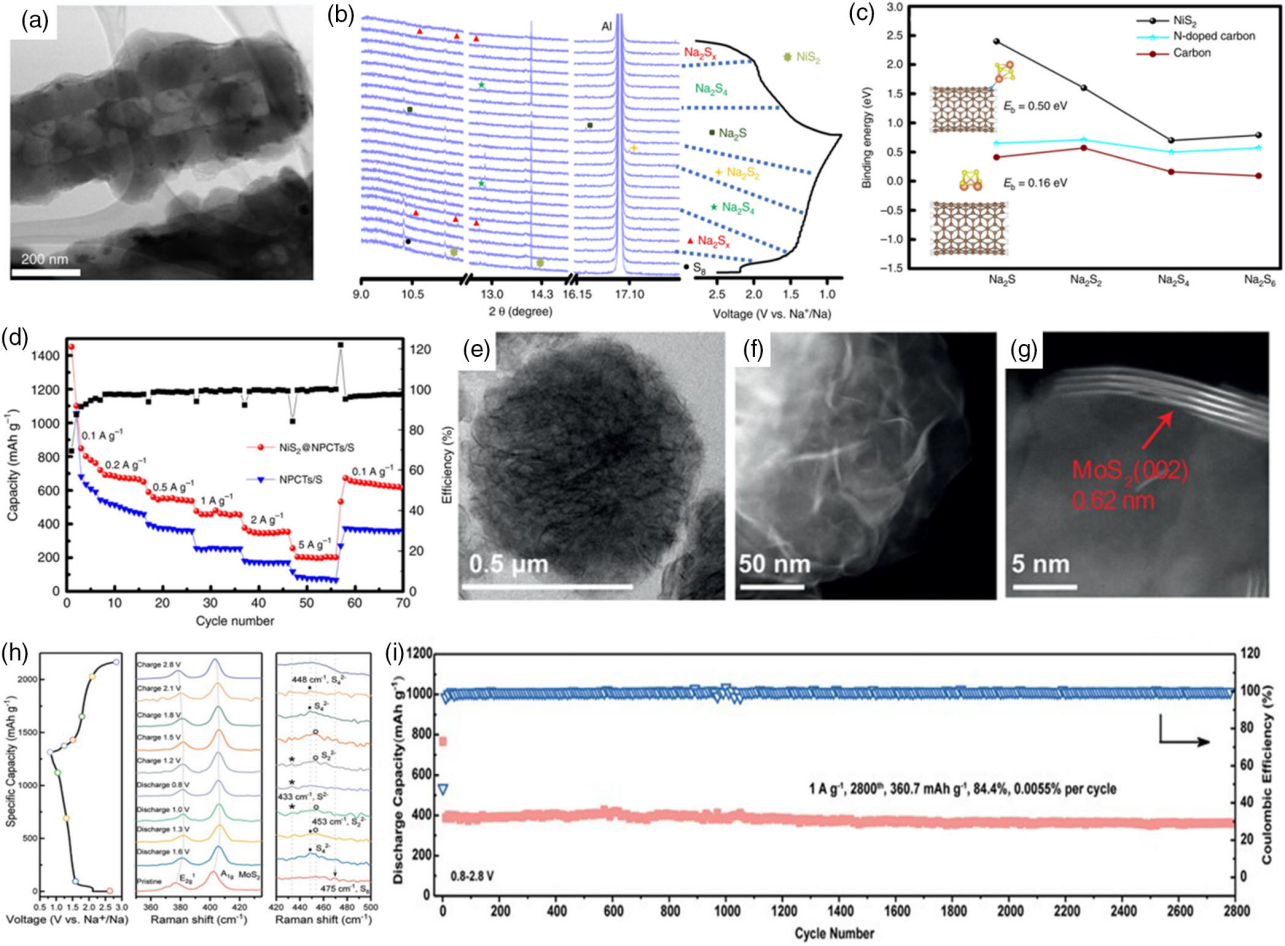
a) STEM image of the NiS_2_@NPCTs/S composite; b) in situ synchrotron XRD patterns of NiS_2_@NPCTs/S electrode during the discharge/charge process at 200 mA g^−1^; c) the binding energies of the Na_2_S_*x*_ on NiS_2_, N‐doped carbon nanotube and carbon nanotube; d) rate performance of the NiS_2_@NPCTs/S cathode; a–d) Reproduced under the terms of the CC‐BY 4.0 license.^[^ ^38^ ^]^ Copyright 2019, The Authors, published by Springer Nature. e,f) TEM and g) HRTEM images of the S/MoS_2_/NCS sample; h) ex situ Raman spectra of the S/MoS_2_/NCS cathode in RT Na–S batteries; i) long‐term cycling performance of the S/MoS_2_/NCS cathode at 1 A g^−1^. e–i) Reproduced with permission.^[^ ^72^ ^]^ Copyright 2021, Wiley‐VCH.

Compared with metal oxides, the relatively good conductivity enables the transition‐metal sulfides as more suitable catalysts in RT Na–S batteries. Especially, some transition‐metal sulfides even exhibit metallic or half‐metallic properties.^[^ ^69^ ^]^ Therefore, it is feasible for the integration of polar transition‐metal sulfides into conductive porous carbon matrix as host for RT Na–S batteries. In recent study, various transition‐metal sulfides have been successfully integrated with porous carbon matrix to realize the efficient adsorption of long‐chain NaPSs and the acceleration of conversion kinetics of NaPSs to (di)sulfides (or the inverse process). Yan et al.^[^ ^70^ ^]^ designed a high kinetics catalyst of hierarchical porous carbon host, i.e., FeS_2_ nanograins in situ grown in the nanocages assembling into microspheres (FeS_2_@NCMS/S) through the spray‐drying process. A shown in Figure 6e,f, the hierarchical porous carbon composed of interconnected nanocages would accommodate the volume variation and physically impede the dissolution of soluble long‐chain NaPSs, as well as facilitate faster charge transfer along the carbon skeleton. Moreover, to deeply explore the catalysis mechanism and catalysis effect of FeS_2_ nanograins, CV, in situ synchrotron XRD (Figure 6g) and in situ TEM were used. Based on these calculations, the authors proposed a two‐step electrocatalytic mechanism with Na_2_S_2_ as an intermediate, which was subsequently reduced to the final Na_2_S product. DFT calculation further demonstrates that the Na_2_S_2_ intermediate has the fastest conversion reaction rate assisted with FeS_2_ catalysis. What's more, the FeS_2_ also has fast sodium‐ion diffusion kinetics. In situ selected area electron diffraction (SAED) patterns confirm the trace of Na_*x*_FeS_2_ with high polaron mobility, which accounts for the small sodium‐ion diffusion barrier. As shown in Figure 6h,i, the hierarchical microporous carbon and the efficient FeS_2_ adsorption–catalysis activity endow the unique sulfur cathode to achieve excellent rate performance (even up to 340 mAh g^−1^ at 2 A g^−1^). At the same time, the large‐scale spray‐drying technique facilitates the mass production of RT Na–S batteries by Yan et al.^[^ ^38^ ^]^ They further investigated the catalysis effect of NiS_2_ in RT Na–S batteries through the implant of NiS_2_ nanocrystals of around 8.3 nm into the 1D nitrogen‐doped carbon nanotubes (NiS_2_@NPCTs/S, **Figure** **7**a). In this system, as observed in the in situ synchrotron XRD patterns (Figure 7b) and UV–vis spectra, the heteroatoms doping of nitrogen as well as the NiS_2_ nanograins show strong adsorption capability towards NaPSs and serve as effective electrocatalytic sites to facilitate the fast reduction of NaPSs to Na_2_S. In addition, as shown in Figure 7c, DFT calculation shows the strong binding energy of Na_2_S on NiS_2_ nanocrystal, indicating the fast conversion kinetics from Na_2_S_4_ to Na_2_S. Based on the aforementioned integrated structural design, a high specific capacity of 401 mAh g^−1^ was maintained after 750 cycles at 1 A g^−1^ and excellent rate performance with 203 mAh g^−1^ at the high current density of 5 A g^−1^ (Figure 7d) was achieved.

In addition, Xu and coworkers^[^ ^71^ ^]^ reported the construction of sodium polysulfides defense system with MoS_2_ nanosheets decorated on the hollow carbon spheres and coated on the glass fiber as barrier layer. The voids of the hollow carbon matrix could serve as the sulfur nanocontainer to physically confine the soluble sulfur species, improve the electronic conductivity as well as accommodate huge volume variations during the evolution process of NaPSs. Moreover, ex situ UV–vis absorption and XPS test indicate the high adsorption capability of polar MoS_2_, which could effectively impede the dissolution of NaPSs, thus alleviating the severe shuttle effect. When used as cathode for RT Na–S batteries, the high reversible capacity of 1090 mAh g^−1^ and stable long cycling performance with slight decrease could be achieved. In the latest study,^[^ ^72^ ^]^ a 3D microﬂower architecture with MoS_2_ nanosheets wrapped on the nitrogen‐doped carbon spheres (S/MoS_2_/NCS, Figure 7e–g) was fabricated and used as sulfur host. Beyond the adsorption capability of polar MoS_2_ to NaPSs, this study proposed that the MoS_2_ nanosheets play a key role in catalyzing the fast conversion from long‐chain NaPSs to the final Na_2_S product. Surprisingly, by tuning the cut‐off voltage from 1.0 to 0.8 V, the designed S/MoS_2_/NCS cathode delivers higher reversible capacity and a pre‐eminent rate capability, which ascribes to the fast conversion kinetics without the formation of high‐order polysulfides. To make deep understanding on this unique sulfur cathode, ex situ Raman spectroscopy and XPS were used. Ex situ Raman spectroscopy results in Figure 7h show the reversible redox reaction process. In addition, XPS results also confirm the critical role of Na_*x*_MoS_2_ in boosting the conversion kinetics. DFT calculations presented that both MoS_2_ and Na_*x*_MoS_2_ show higher binding energy than pure carbon matrix, indicating the stronger adsorption capability for sulfur species. Both the MoS_2_ and Na_*x*_MoS_2_ active sites show relatively higher binding energy with Na_2_S, which effectively promotes the nucleation of Na_2_S, leading to a fast electrocatalysis conversion kinetics. Due to the synergistic effect of MoS_2_ and Na_*x*_MoS_2_ active sites, the impressive long cycle life with 360.7 mAh g^−1^ after 2800 cycles at the high current density of 1 A g^−1^ was maintained (Figure 7i) and pre‐eminent rate capability with even up to 470.7 mAh g^−1^ at 5.0 A g^−1^ could be realized.

**Figure 8:**
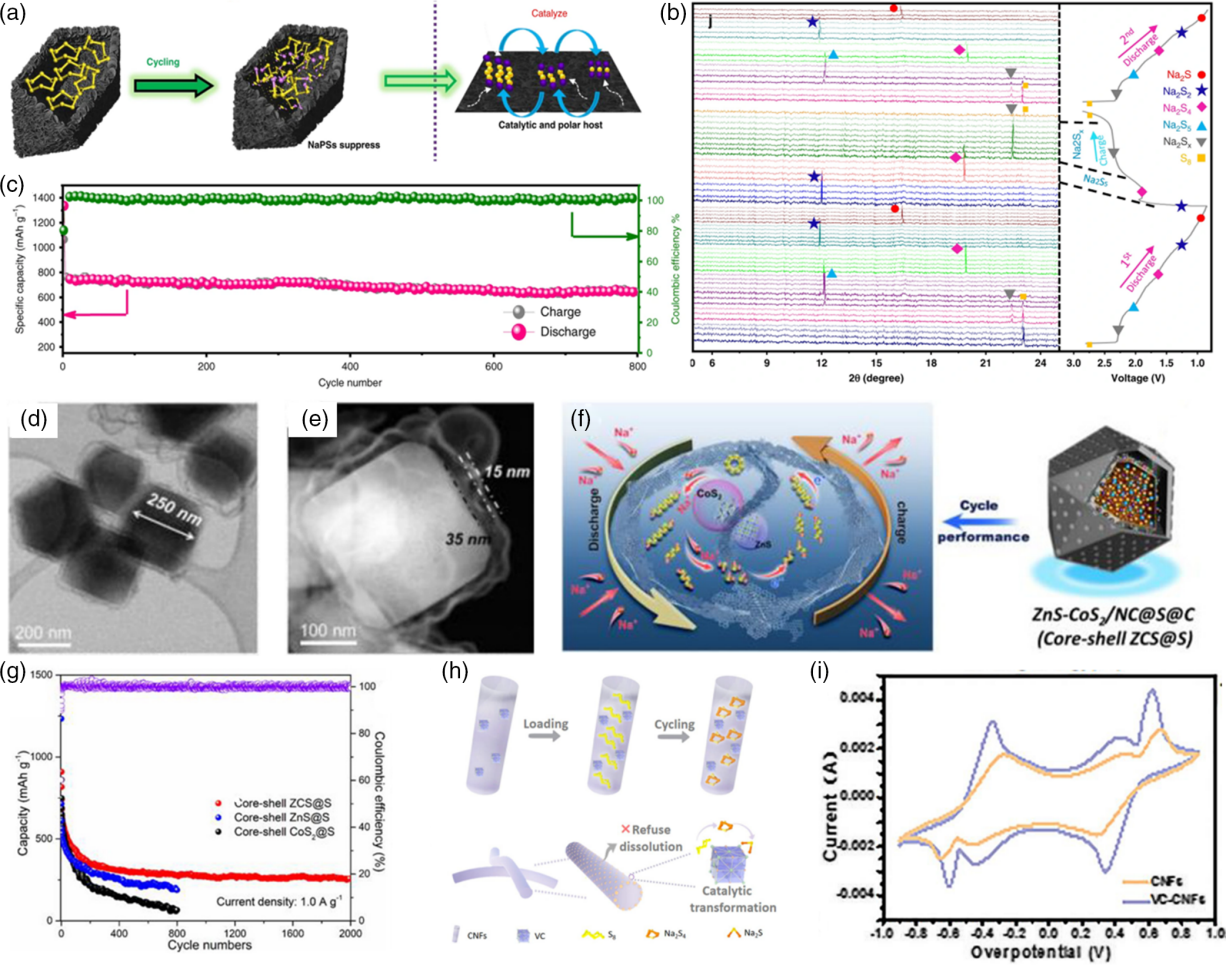
a) Schematic illustration of the structural design advantages of the S@BPCS cathode in suppressing the NaPSs; b) in situ XRD patterns of the S@BPCS cathode during the first two discharge/charge process; c) long cycling performance of the S@BPCS cathode; a–c) Reproduced under the terms of the CC‐BY 4.0 license.^[^ ^73^ ^]^ Copyright 2020, The Authors, published by Springer Nature. d) TEM and e) high angle annular dark field (HAADF) images of the core–shell ZCS@S cathode; f) the structural advantages of the ZCS@S cathode during the charge/discharge process; g) The long cycling process of the ZCS@S cathode at 1 A g^−1^; d–g) Reproduced with permission.^[^ ^74^ ^]^ Copyright 2020, American Chemical Society. h) Schematic illustration of the synthetic process of the VC‐CNFs@S composite; i) The CV profiles of the symmetric cells for the VC‐CNFs composite. h,i) Reproduced with permission.^[^ ^43^ ^]^ Copyright 2020, Elsevier.

Similarly, Xu and coworkers^[^ ^73^ ^]^ further investigated the adsorption–catalysis effect of CoS_2_ as sulfur host in RT Na–S batteries. As shown in **Figure** **8**a, the internal hollow bipyramid prisms CoS_2_/C architecture with interwoven surfaces and uniform slit‐like micropores has been fabricated and used as polar sulfur host (S@BPCS). The integrated structure design as well as the polar nature of CoS_2_ could synergistically realize physical confinement of sulfur species, alleviation volumetric expansion as well as chemical adsorption. In addition, the electrocatalysis mechanism was further evaluated via the advanced in situ/ex situ characterizations. Ex situ XPS results indicate the strong chemical interaction between BPCS and NaPSs. Combining with in situ XRD (Figure 8b), in situ Raman and ex situ high resolution transmission electron microscopy (HRTEM)/SAED results, no Na_2_S and Na_2_S_5_ were detected, indicating the efficient catalysis effect of polar CoS_2_ with fast conversion rate of short‐chain NaPSs to long‐chain NaPSs. As a result, S@BPCS cathode delivers ultrastable long cycling life with remaining 701 mAh g^−1^ at a high current rate of 0.5 C after 350 cycles (Figure 8c). A high mass loading in sulfur cathode would lead to sever shuttle effect with low sulfur utilization and fast capacity decay. Therefore, the electrochemical performance with high sulfur loading is crucial for the large‐scale practical application. Thus, the S@BPCS cathode with ultrahigh mass loading of 7.3 and 9.1 mg cm^−2^ was examined, which shows stable cycling performance over 70 cycles at 0.5 C almost without capacity decay.

Apart from the single‐component catalyst for the adsorption of long‐chain NaPSs and catalyzing the fast redox reaction kinetics, the multiple catalytically active sites with more enhanced adsorption and catalytic effect hold significant promise. As shown in Figure 8d,e, Liu et al.^[^ ^74^ ^]^ designed an electrocatalyzing sulfur cathode with hierarchical porous core–shell carbonaceous structure and ZnS/CoS_2_ multisulfiphilic sites (core–shell ZCS@S) for RT Na–S batteries. The rational structure design of this sulfur host displays the following merits (Figure 8f). First, the mesoporous carbonaceous core derived from metal–organic frameworks would guarantee the high sulfur loading as well as the large surface between active material and the carbon host. In addition, the microporous carbon shell could physically prevent the dissolution of long‐chain NaPSs into electrolyte. What's more, the inner space between the core–shell structure could effectively accommodate the volume change arising from the evolution of sulfur species during the charge/discharge process, thus enabling a much stable long cycling life. The UV–vis spectra and the charging/discharging voltage profiles (first cycle) of the Na_2_S cathode indicate that the multisulfiphilic sites exhibit the enhanced adsorption capability of NaPSs and reduced energy barrier from Na_2_S to long‐chain NaPSs during charge process. In situ synchrotron XRD and XPS analysis demonstrate that the catalysis could be improved with the increase in sulfiphilic sites on CoS_2_ and ZnS. To get deep atomic insight into the adsorption and catalysis effect of CoS_2_ and ZnS, DFT calculation was carried out. Both the CoS_2_ and ZnS active sites show the synergistic effect on NaPSs immobilization and the kinetics of conversion reaction. Compared with the single CoS_2_ or ZnS decorated sulfur host, the core–shell ZCS@S cathode delivers very stable long cycling performance over 2000 cycles (Figure 8g).

#### 4.2.3 Transition‐Metal Carbides

The vanadium carbide nanoparticles wrapped within the carbon nanofibers as sulfur host (VC‐CNFs@S) with confining, trapping and catalyzing effects for RT Na–S battery were further investigated by Xu et al. (Figure 8h).^[^ ^43^ ^]^ The XPS and UV–vis absorption results after adsorbed in the Na_2_S_6_ solution demonstrate the strong chemical interaction between the VC–CNFs polar matrix and the polysulfides, which is directly evidenced by the possible electrons transformation between Na_2_S_6_ and V atoms. In addition, the catalytic activity of the polar VC was further evaluated via the CV test of the symmetric cells in 0.2 m Na_2_S_6_ electrolyte shown in Figure 8i. Obviously, the VC‐CNFs matrix exhibits prominent current response, indicating the enhanced catalytic effect with fast conversion kinetics. When used as cathode for RT Na–S batteries, a stable long cycling performance with a high capacity retention of 96.2% over 2000 cycles was achieved.

### 4.3 Heterostructure Catalysts

**Figure 9:**
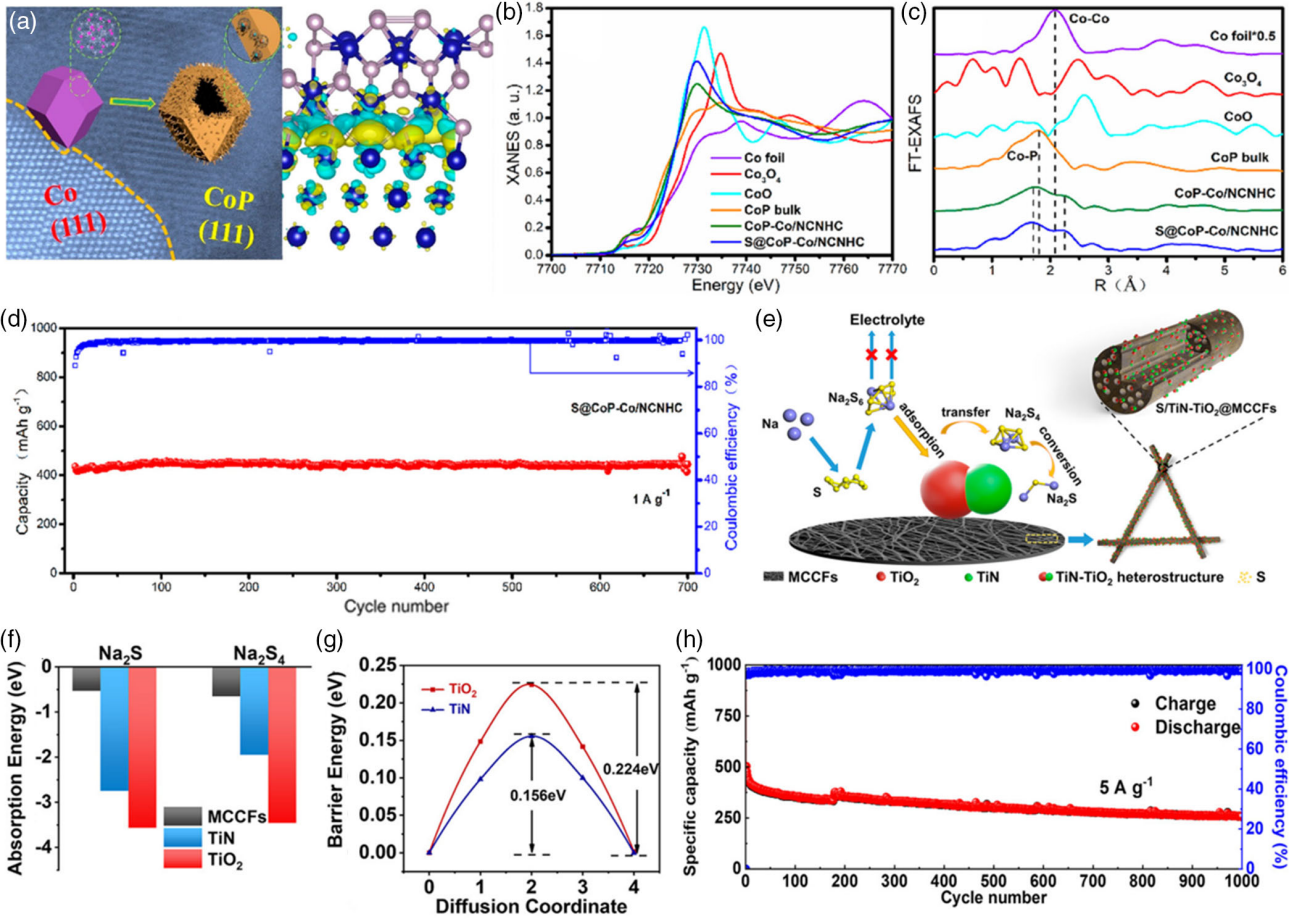
a) Schematic illustration and HRTEM image of the S@CoP‐Co/NCNHC composite; b) XANES and c) FT‐EXAFS spectra of the Co K‐edge in the S@CoP‐Co/NCNHC, CoP‐Co/NCNHC, CoP, CoO, Co_3_O_4_, and Co foil; d) long cycling performance of the S@CoP‐Co/NCNHC cathode at 1 A g^−1^; a–d) Reproduced with permission.^[^ ^75^ ^]^ Copyright 2020, American Chemical Society. e) Schematic illustration of the structural advantages and mechanism of the TiN‐TiO_2_@MCCFs cathode for RT Na–S batteries; f) binding energy values and g) energy profiles of Na_2_S/Na_2_S_4_ and Na^+^ on TiN and TiO_2_ surfaces, respectively; h) long cycling performance of the TiN‐TiO_2_@MCCFs cathode at 5 A g^−1^. e–h) Reproduced with permission.^[^ ^44^ ^]^ Copyright 2021, American Chemical Society.

As aforementioned, single‐component catalyst has been widely used in RT Na–S batteries. More specifically, the introduction of secondary catalyst component, namely “heterostructure catalysts,” which refers to composites composed of two or more components that are chemically bonded, could effectively incorporate the synergistic effects of each component, providing an effective strategy to address the intrinsic issues of RT Na–S batteries. Therefore, building heterostructure‐decorated porous carbon matrix as sulfur host plays vital role in synchronous realizing anchoring of long‐chain NaPSs and accelerating of redox kinetics. In recent study, Yan et al.^[^ ^75^ ^]^ successfully fabricated the CoP–Co heterostructures in situ confined in the nitrogen‐doped porous carbon nanocages interpenetrated with carbon nanotubes as sulfur host (S@CoP‐Co/NCNHC), which was derived from the heat treatment of MOF. As shown in **Figure** **9**a, the multiregion Janus‐featured CoP–Co heterostructures could be detected by the HAADF–STEM image with clear lattice interface between two different d‐spacings of 0.249 and 0.205 nm, belonging to the (111) planes of CoP and Co, respectively. The electron energy loss spectroscopy (EELS) spectra of the Co and P L‐edges confirm the coexistence of CoP and metal Co. XANES and extended X‐ray absorption fine structure (EXAFS) spectra in Figure 9b,c further confirm the existence of Co and CoP heterostructure, which adjusts the electronic structure of phosphorus and cobalt atoms, hence leading to the improved electronic conductivity and electrocatalytic activity. The theoretical calculation results reveal the enhanced adsorption energy between the CoP–Co heterostructure and the Na_2_S_*x*_ clusters. Meanwhile, the densities of states (DOS) of CoP–Co exhibit a higher Fermi level and a larger bandwidth, demonstrating the easy charge transfer from CoP–Co surface to Na_2_S_*x*_. Benefiting from the synergistic effect of the CoP electrocatalyst and the metal Co conductive support, as well as the CoP–Co heterostructures and the rational‐designed porous carbon matrix, the S@CoP‐Co/NCNHC cathode delivers excellent rate capability and much stable long cycling performance with 448 mAh g^−1^ after 700 cycles under the higher current density of 1 A g^−1^ (Figure 9d).

In addition, as shown in Figure 9e, Zheng and coworkers^[^ ^44^ ^]^ reported the unique TiN–TiO_2_ heterostructures with impressive adsorption–diffusion–catalysis effects in situ wrapped in the multichannel porous carbon matrix as sulfur host for RT Na–S batteries (TiN–TiO_2_@MCCFs). Clear lattice interface between TiN and TiO_2_ is clearly visible in the HRTEM images. In addition, the generation of TiN–TiO_2_ heterostructure could be proved via XRD and Raman test with enhanced electronic conductivity. The first‐principles calculation analysis of adsorption energies and energy barrier on TiO_2_ and TiN proves the adsorption–diffusion–catalysis effects (i.e., TiO_2_ chemiadsorption, TiN electrocatalysis as well as the interface diffusion) of the TiN–TiO_2_ heterostructure. Moreover, the adsorption effect of the TiN–TiO_2_ heterostructure was evaluated via the visualized adsorption tests of Na_2_S_6_. Obviously, solution containing TiN–TiO_2_ heterostructures and TiO_2_ exhibits rather light color, indicating the stronger adsorption capability of the polar TiO_2_ component toward soluble NaPSs. In addition, to further investigate the superiority of TiN–TiO_2_ heterostructure with respect to the reduction process, the precipitation experiments of Na_2_S_6_ exhibit that the current density and Na_2_S precipitation capacity of the TiN–TiO_2_@MCCFs sample are higher than those of bare TiO_2_ matrix, indicating the stronger electrocatalytic conversion kinetics promoted by TiN. Benefiting from the strong adsorption and electrocatalysis effectiveness arising from the TiN–TiO_2_ heterostructure, NaPSs are first adsorbed on TiO_2_ site, and then transferred to TiN through the TiN–TiO_2_ interface, facilitating the fast electrocatalysis to low‐order insoluble short‐chain sodium sulfides, thereby alleviating the NaPSs shuttling effects and promoting the redox kinetics. The adsorption and catalysis activity could be also confirmed by the DFT calculation (Figure 9f,g). Consequently, due to the TiN–TiO_2_ heterostructure and the multichannel porous carbon matrix, impressive rate capability with the ultrahigh reversible capacity of 440.2 mAh g^−1^ at 5 A g^−1^ and superior long life span with 257.1 mAh g^−1^ at 5 A g^−1^ after 1000 cycles could be achieved (Figure 9h).

## 5 Other Types of Interaction with Adsorption–Catalysis Effects

### 5.1 Lewis Acid–Base Interaction

**Figure 10:**
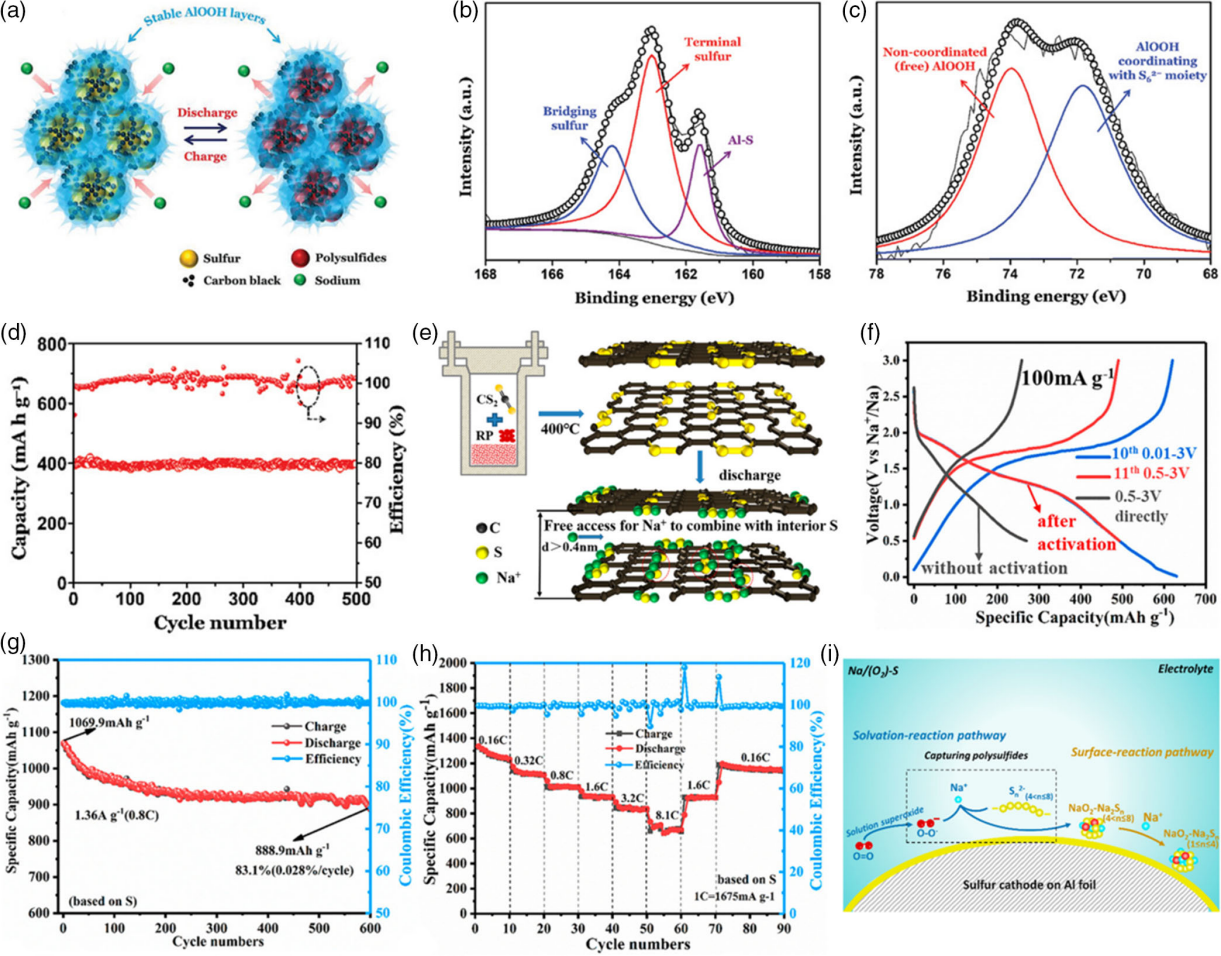
a) Schematic illustration of the structure stability assisted with the AlOOH layer; b) deconvoluted S2p_3/2_ and c) Al_2p_ XPS spectra of polysulfide‐adsorbed AlOOH sample; d) long‐term cycling of the S@CB@AlOOH cathode at 1 C; a–d) Reproduced with permission.^[^ ^76^ ^]^ Copyright 2020, Wiley‐VCH. e) Schematic illustration of the structural advantages and mechanism of the covalent‐SC cathode in RT Na–S batteries; f) voltage profiles of the covalent‐SC electrode at different voltage window; g) Long cycle performance and h) rate capability of the covalent‐SC cathode; e–h) Reproduced with permission.^[^ ^78^ ^]^ Copyright 2020, American Chemical Society. i) Schematic illustration of the effect of sulfur–oxygen chemistry on the discharge process. Reproduced with permission.^[^ ^79^ ^]^ Copyright 2020, American Chemical Society.

Mitra and coworkers^[^ ^76^ ^]^ innovatively designed the AlOOH nanosheets in situ grown on the sulfur/carbon black composite (S@CB@AlOOH) to realize the structural immobilization and chemical adsorption for effectively suppressing the dissolution and shuttle of NaPSs in RT Na–S batteries. As shown in **Figure** **10**a, 3D interconnected thin AlOOH nanosheets are anchored on the sulfur/carbon black composite with the core–shell structure. The XPS results of the Na_2_S_6_ solution with AlOOH in Figure 10b,c show the chemical interaction between AlOOH and Na_2_S_6_, by the formation of Al—S bond. To get atomic insight into the chemical interaction between AlOOH additive and the intermediates, DFT calculation was conducted. Obviously, AlOOH shows gradually enhanced binding energy between the long‐chain NaPSs and Na_2_S, indicating the strong chemical interaction and catalysis effect with enhanced reduction kinetics. Moreover, Mulliken charge distribution combining with the DOS results confirm the existence of the Lewis acid–base interaction between AlOOH and Na_2_S, hence opening a new opportunity for the design of advanced polar sulfur host for RT Na–S batteries. Especially, as shown in Figure 10d, S@CB@AlOOH demonstrates an impressive long cycling stability with 378 mAh g^−1^ after 500 cycles (96% capacity retention) at the current density of 1 A g^−1^.

### 5.2 Covalent Sulfur–Carbon Cathode

The construction of covalent sulfur–carbon cathode with covalent S—C chemical bonds could effectively strengthen the electronic contact of active sulfur and carbon matrix with improved electronic conductivity. Moreover, the covalent sulfur–carbon cathode could also induce the formation of short‐chain NaPSs instead of long‐chain NaPSs, thus effectively alleviating the severe shuttle effect. To effectively alleviate the shuttle effect by suppressing the dissolution of long‐chain NaPSs, the controllable chain‐length of intermediate sulfide species during charge/discharge process may fundamentally solve the challenge. As observed in Figure 10e, Ji and coworkers^[^ ^77^ ^]^ developed a novel 3D coral‐like covalent sulfur–carbon complex cathode derived from the carbonization of a covalent sulfur–carbon complex (SC‐BDSA). The formed long‐chain sulfur–carbon‐ridged complex can be electrically activated at lower voltage state and served as a capacity sponsor with reduced long‐chain NaPSs shuttle effect (Figure 10f). Moreover, in recent study,^[^ ^78^ ^]^ a novel sulfur cathode has been innovatively designed with the in situ formation of covalent sulfur–carbon composite (covalent‐SC) during the wet‐chemical solvothermal process assisted with CS_2_ and red phosphorus. Various tests, including XRD, Raman, Fourier transform infrared spectroscopy (FTIR) spectroscopy, XPS and thermogravimetric analysis (TG) analysis, made a deep comprehensive understanding on the existence form of sulfur active, showing the formation of covalent sulfur–carbon composite. Different from the conventional sulfur–carbon cathodes, the in situ formed covalent sulfur–carbon composite could tightly fix sulfur on molecular level, endowing much enhanced electronic conductivity and high sulfur utilization as well as avoiding the shuttle effect with the formation of low‐order sulfides, which accounts for the outstanding electrochemical performance. The conventional sulfur–carbon bond could be electrochemically broken at lower potential. As shown in Figure 10g,h, when used in RT Na–S batteries, high discharge capability of 888.9 and 811.4 mAh g^−1^ could be achieved after 600 and 950 cycles at 0.8 and 1.6 C, respectively. Moreover, covalent‐SC cathode even exhibits an impressively high capacity of 700 mAh g^−1^ at the higher current rate of 8.1 C.

### 5.3 Sulfur–Oxygen Chemistry

The traditional sulfur cathode design for RT Na–S batteries would inevitably induce the challenges of long‐chain NaPSs dissolution and the resulting severe shuttle effect. Surprisingly, Li and coworkers^[^ ^79^ ^]^ proposed that altering the reaction pathway via a new sulfur–oxygen chemistry with effective adsorption activity could realize the desirable electrochemical performance for RT Na–S batteries. In this study, the incorporation of extra oxygen dissolved in electrolyte with the formation of NaO_2_–Na_2_S_*n*_ (1 < *n* ≤ 8) clusters could effectively suppress the dissolution of long‐chain NaPSs and alleviate shuttle effect, leading to much enhanced reversible capacity and long‐term cycling stability (Figure 10i).

## 6 Conclusion

This Review systematically summarizes the working mechanisms and challenges in RT Na–S batteries and highlights recent advances of effective modification strategies based on sulfur cathode design. In consequence of the complicated multistep reaction process, the practical application of RT Na–S batteries mainly faces the obstacles of low specific capacity and unsatisfying long cycling life span due to the sluggish reaction kinetics, huge volume variation as well as severe shuttle effect of the sulfur cathode. As the obstacle of RT Na–S batteries mainly comes from the sulfur cathode, the optimization of the sulfur host holds significant promise. First, the design of novel hierarchical porous carbon host with high surface area and high porosity could endow the improved electronic conductivity with fast electron/charge transfer, accommodate the sulfur active material with high loading and alleviate volumetric swelling, as well as physically confine the dissolution of long‐chain NaPSs. Then, the integration of electrocatalytic active materials with strong adsorption and catalysis activity toward NaPSs into the rational‐designed porous carbon matrix is confirmed to be an effective strategy. Moreover, it is crucial to optimize the electrocatalyst structure with high electronic conductivity, well‐designed nanosized structure, efficient adsorption, and catalytic activity to realize the fully exposing active sites and expediting ion transport. Based on the aforementioned summary, the design principles of highly efficient sulfur matrix in RT Na–S batteries should be developed in the direction of the “confining–adsorption–catalyzing” synergistic effects.

Although advanced achievements for RT Na–S batteries have been realized by the integrated host design with “confining–adsorption–catalyzing” effect, several challenges still remain and should be considered.

### 6.1 In‐Depth Exploration of Catalytic Mechanism and Design Principles of High‐Efficiency Catalysts

Commonly, the well‐designed catalysts with desirable adsorption effect toward NaPSs and high catalytic capability are indirectly convinced by the enhanced adsorption energy and small Gibbs free energy via theoretical calculations. However, there is still a lack of deep understanding in the ion/electron transfer and conversion at the working surface/interface of electrocatalyst. For example, what is the charge transfer path on the catalysis surface during the redox process? Therefore, getting deep insight into the mechanism of the catalytic effect still has a long way to go, which can draw lessons from other fields, such as Li–S batteries, electrocatalytic reduction of CO_2_ or electrolysis of water. Furthermore, finding a balance between the “adsorption–catalysis” function is essential to realize high sulfur utilization and fast kinetics via the effective adsorption, release, and conversion of polysulfides. For the design of high‐efficiency electrocatalyst, the construction of multifunctional heterostructure electrocatalyst with strong adsorption and catalysis effect to simultaneously catalyze the reduction and oxidation processes is regarded as a very promising strategy. Moreover, engineering the polarity and electronic structure of the heterostructures may realize a smooth “adsorption–diffusion–conversion” effect for highly efficient NaPS management. In addition, the amount of catalysis should be considered due to the sacrifices of the overall energy density of RT Na–S batteries. Therefore, engineering the adsorptive/catalytic sites with well dispersed and nanosized structure on the rational‐designed carbon host with high specific surface area and porosity could effectively increase the utilization of active sites.

### 6.2 The Development of Advanced In Situ/Ex Situ Characterization

The mechanistic studies on the fundamental reaction mechanism of the catalytic activities for RT Na–S batteries are very urgent. Hence, related electrochemical characterization should be carried out, such as CV of symmetrical battery, the nucleation, and decomposition experiments of Na_2_S, as well as drawing lessons from the electrocatalysis fields with exchange current density or linear sweep voltammetry (LSV) measurements, synergisticly reflecting the effectiveness and mechanism of catalysis. In addition, in situ characterization should be developed to real‐time monitor the instantaneous change and evolution process inside the battery during the operation process, providing accurate guidance for the design of advanced RT Na–S batteries.

### 6.3 Challenges for Practical Applications: High Sulfur Loading and Low Electrolyte/Sulfur Ratio

High sulfur content in the cathode and high areal mass loading as well as low electrolyte/sulfur (*E*/*S*) ratio have profound inﬂuence on the speciﬁc energy density of RT Na–S batteries, which can significantly determine the practical applications. In this respect, the introduction of highly effective electrocatalysts into the sulfur host under the lean electrolyte and high areal sulfur loading conditions shows great research value.

With many challenges and opportunities, the RT Na–S batteries show significant promise in large‐scale energy storage system due to the high energy density. The rational design of sulfur cathode, sodium anode as well as the matched electrolyte still has a long way to go and would arouse extensive research interest in the near future.

## Conflict of Interest

The authors declare no conflict of interest.

## References

[1] J.‐Y. Hwang , S.‐T. Myung , Y.‐K. Sun , Chem. Soc. Rev. 2017, 46, 3529. 28349134 10.1039/c6cs00776g

[2] M. Li , J. Lu , Z. Chen , K. Amine , Adv. Mater. 2018, 30, 1800561. 10.1002/adma.201800561 29904941

[3] J. B. Goodenough , Acc. Chem. Res. 2013, 46, 1053. 22746097 10.1021/ar2002705

[4] J.‐M. Tarascon , M. Armand , Materials for Sustainable Energy: A Collection of Peer-Reviewed Research and Review Articles from Nature Publishing Group, nature publishing group, 2011, pp. 171–179.

[5] J. B. Goodenough , K.‐S. Park , J. Am. Chem. Soc. 2013, 135, 1167. 23294028 10.1021/ja3091438

[6] D. Gong , C. Wei , Z. Liang , Y. Tang , Small Sci. 2021, 1, 202100014. 10.1002/smsc.202100014 PMC11935890 40212712

[7] F. Liu , Z. Zhang , S. Ye , Y. Yao , Y. Yu , Acta Phys. Chim. Sin. 2020, 37, 2006021.

[8] C. Zhang , F. Wang , J. Han , S. Bai , J. Tan , J. Liu , F. Li , Small Struct. 2021, 2, 202100009.

[9] A. Manthiram , S. H. Chung , C. Zu , Adv. Mater. 2015, 27, 1980. 25688969 10.1002/adma.201405115

[10] D. Aurbach , B. D. McCloskey , L. F. Nazar , P. G. Bruce , Nat. Energy 2016, 1, 1.

[11] S. Wei , S. Xu , A. Agrawral , S. Choudhury , Y. Lu , Z. Tu , L. Ma , L. A. Archer , Nat. Commun. 2016, 7, 1. 10.1038/ncomms11722 PMC4906167 27277345

[12] X. Zeng , J. Li , L. Liu , Renewable Sustainable Energy Rev. 2015, 52, 1759.

[13] T. H. Hwang , D. S. Jung , J.‐S. Kim , B. G. Kim , J. W. Choi , Nano Lett. 2013, 13, 4532. 23981085 10.1021/nl402513x

[14] X. Lu , B. W. Kirby , W. Xu , G. Li , J. Y. Kim , J. P. Lemmon , V. L. Sprenkle , Z. Yang , Energy Environ. Sci. 2013, 6, 299.

[15] S. Zheng , P. Han , Z. Han , P. Li , H. Zhang , J. Yang , Adv. Energy Mater. 2014, 4, 1400226.

[16] Y. X. Wang , B. Zhang , W. Lai , Y. Xu , S. L. Chou , H. K. Liu , S. X. Dou , Adv. Energy Mater. 2017, 7, 1602829.

[17] Q. Guo , Z. Zheng , Adv. Funct. Mater. 2020, 30, 1907931.

[18] H. Ryu , T. Kim , K. Kim , J.‐H. Ahn , T. Nam , G. Wang , H.‐J. Ahn , J. Power Sources 2011, 196, 5186.

[19] Y. Song , W. Cai , L. Kong , J. Cai , Q. Zhang , J. Sun , Adv. Energy Mater. 2020, 10, 1901075.

[20] P. Wang , B. Xi , M. Huang , W. Chen , J. Feng , S. Xiong , Adv. Energy Mater. 2021, 11, 2002893.

[21] M. Zhang , W. Chen , L. Xue , Y. Jiao , T. Lei , J. Chu , J. Huang , C. Gong , C. Yan , Y. Yan , Adv. Energy Mater. 2020, 10, 1903008.

[22] T. Tang , Y. Hou , Small Methods 2020, 4, 1900001.

[23] S. Huang , Z. Wang , Y. Von Lim , Y. Wang , Y. Li , D. Zhang , H. Y. Yang , Adv. Energy Mater. 2021, 11, 2003689.

[24] L. Zhou , D. L. Danilov , R. A. Eichel , P. H. Notten , Adv. Energy Mater. 2021, 11, 2001304.

[25] L. Du , H. Wang , M. Yang , L. Liu , Z. Niu , Small Struct. 2020, 1, 202000047.

[26] A. Manthiram , X. Yu , Small 2015, 11, 2108. 25565554 10.1002/smll.201403257

[27] Y. X. Wang , W. H. Lai , S. L. Chou , H. K. Liu , S. X. Dou , Adv. Mater. 2020, 32, 1903952. 10.1002/adma.201903952 31566255

[28] D. Liu , Z. Li , X. Li , Z. Cheng , L. Yuan , Y. Huang , ChemPhysChem 2019, 20, 3164. 31553116 10.1002/cphc.201900595

[29] Y. Wang , D. Zhou , V. Palomares , D. Shanmukaraj , B. Sun , X. Tang , C. Wang , M. Armand , T. Rojo , G. Wang , Energy Environ. Sci. 2020, 13, 3848.

[30] S. Zhang , Y. Yao , Y. Yu , ACS Energy Lett. 2021, 6, 529.

[31] X. Yu , A. Manthiram , Adv. Funct. Mater. 2020, 30, 2004084.

[32] Y.‐X. Wang , J. Yang , W. Lai , S.‐L. Chou , Q.‐F. Gu , H. K. Liu , D. Zhao , S. X. Dou , J. Am. Chem. Soc. 2016, 138, 16576. 27992193 10.1021/jacs.6b08685

[33] J. Mou , T. Liu , Y. Li , W. Zhang , M. Li , Y. Xu , J. Huang , M. Liu , J. Mater. Chem. A 2020, 8, 24590.

[34] H. Li , M. Zhao , B. Jin , Z. Wen , H. K. Liu , Q. Jiang , Small 2020, 16, 1907464. 10.1002/smll.201907464 32548956

[35] J. Song , T. Xu , M. L. Gordin , P. Zhu , D. Lv , Y. B. Jiang , Y. Chen , Y. Duan , D. Wang , Adv. Funct. Mater. 2014, 24, 1243.

[36] A. Douglas , R. Carter , L. Oakes , K. Share , A. P. Cohn , C. L. Pint , ACS Nano 2015, 9, 11156. 26529682 10.1021/acsnano.5b04700

[37] B.‐W. Zhang , T. Sheng , Y.‐D. Liu , Y.‐X. Wang , L. Zhang , W.‐H. Lai , L. Wang , J. Yang , Q.‐F. Gu , S.‐L. Chou , Nat. Commun. 2018, 9, 1. 30287817 10.1038/s41467-018-06144-x PMC6172263

[38] Z. Yan , J. Xiao , W. Lai , L. Wang , F. Gebert , Y. Wang , Q. Gu , H. Liu , S.‐L. Chou , H. Liu , Nat. Commun. 2019, 10, 1. 31641115 10.1038/s41467-019-11600-3 PMC6805862

[39] A. Y. S. Eng , V. Kumar , Y. Zhang , J. Luo , W. Wang , Y. Sun , W. Li , Z. W. Seh , Adv. Energy Mater. 2021, 11, 2003493.

[40] Q. Wang , C. Cai , M. Dai , J. Fu , X. Zhang , H. Li , H. Zhang , K. Chen , Y. Lin , H. Li , J. Hu , M. Miyauchi , M. Liu , Small Sci. 2020, 1, 202000028.

[41] Y. Cui , Z. Cao , Y. Zhang , H. Chen , J. Gu , Z. Du , Y. Shi , B. Li , S. Yang , Small Sci. 2021, 1, 202100017. 10.1002/smsc.202100017 PMC11936024 40212711

[42] J. Yang , W. Li , D. Wang , Y. Li , Small Struct. 2020, 2, 202000051.

[43] W. Tang , W. Zhong , Y. Wu , Y. Qi , B. Guo , D. Liu , S.‐J. Bao , M. Xu , Chem. Eng. J. 2020, 395, 124978.

[44] X. Ye , J. Ruan , Y. Pang , J. Yang , Y. Liu , Y. Huang , S. Zheng , ACS Nano 2021, 15, 5639. 33666431 10.1021/acsnano.1c00804

[45] W. Du , Y. Wu , T. Yang , B. Guo , D. Liu , S.‐J. Bao , M. Xu , Chem. Eng. J. 2020, 379, 122359.

[46] H. Liu , W.‐H. Lai , Y. Liang , X. Liang , Z.‐C. Yan , H.‐L. Yang , Y.‐J. Lei , P. Wei , S. Zhou , Q.‐F. Gu , J. Mater. Chem. A 2021, 9, 566.

[47] C. Meng , P. Das , X. Shi , Q. Fu , K. Müllen , Z.‐S. Wu , Small Sci. 2021, 1, 202000076. 10.1002/smsc.202000076 PMC11935899 40213354

[48] Y. X. Wang , B. Zhang , W. Lai , Y. Xu , S. L. Chou , H. K. Liu , S. X. Dou , Adv. Energy Mater. 2017, 7, 1770140.

[49] N. Wang , Y. Wang , Z. Bai , Z. Fang , X. Zhang , Z. Xu , Y. Ding , X. Xu , Y. Du , S. Dou , Energy Environ. Sci. 2020, 13, 562.

[50] S. Zhao , Z. Guo , K. Yan , X. Guo , S. Wan , F. He , B. Sun , G. Wang , Small Struct. 2020, 2, 202000054.

[51] G. He , X. Ji , L. Nazar , Energy Environ. Sci. 2011, 4, 2878.

[52] J. Zhang , D. W. Wang , W. Lv , L. Qin , S. Niu , S. Zhang , T. Cao , F. Kang , Q. H. Yang , Adv. Energy. Mater. 2018, 8, 1801361.

[53] Q. Guo , S. Li , X. Liu , H. Lu , X. Chang , H. Zhang , X. Zhu , Q. Xia , C. Yan , H. Xia , Adv. Sci. 2020, 7, 1903246. 10.1002/advs.201903246 PMC7284216 32537400

[54] Y. Liu , Y. Zhai , Y. Xia , W. Li , D. Zhao , Small Struct. 2021, 2, 202000118.

[55] J. Zhang , J. Han , Q. Yun , Q. Li , Y. Long , G. Ling , C. Zhang , Q.‐H. Yang , Small Sci. 2021, 1, 202000063. 10.1002/smsc.202000063 PMC11935941 40213616

[56] S. Xin , L. Gu , N.‐H. Zhao , Y.‐X. Yin , L.‐J. Zhou , Y.‐G. Guo , L.‐J. Wan , J. Am. Chem. Soc. 2012, 134, 18510. 23101502 10.1021/ja308170k

[57] S. Xin , Y. X. Yin , Y. G. Guo , L. J. Wan , Adv. Mater. 2014, 26, 1261. 24338949 10.1002/adma.201304126

[58] G. Xia , L. Zhang , X. Chen , Y. Huang , D. Sun , F. Fang , Z. Guo , X. Yu , Energy Storage Mater. 2018, 14, 314.

[59] L. Zhang , B. Zhang , Y. Dou , Y. Wang , M. Al-Mamun , X. Hu , H. Liu , ACS Appl. Mater. Interfaces 2018, 10, 20422. 29762005 10.1021/acsami.8b03850

[60] F. Xiao , X. Yang , H. Wang , J. Xu , Y. Liu , D. Y. Yu , A. L. Rogach , Adv. Energy Mater. 2020, 10, 2000931.

[61] F. Jin , B. Wang , J. Wang , Y. Wang , Y. Ning , J. Yang , Z. Zhang , P. Liu , Y. Zhou , D. Wang , Matter 2021, 4, 1768.

[62] B. Guo , W. Du , T. Yang , J. Deng , D. Liu , Y. Qi , J. Jiang , S. J. Bao , M. Xu , Adv. Sci. 2020, 7, 1902617. 10.1002/advs.201902617 PMC7029643 32099760

[63] Q. Ma , W. Zhong , G. Du , Y. Qi , S.‐j. Bao , M. Xu , C. Li , ACS Appl. Mater. Interfaces 2021, 11852. 33656849 10.1021/acsami.0c21267

[64] B. Qiao , J. Liu , L. Allard , A. Wang , Y. Cui , T. Zhang , X. Yang , J. Li , Z. Jiang , Microsc. Microanal. 2012, 18, 350.

[65] B. W. Zhang , T. Sheng , Y. X. Wang , S. Chou , K. Davey , S. X. Dou , S. Z. Qiao , Angew. Chem., Int. Ed. 2019, 58, 1484. 10.1002/anie.201811080 30537071

[66] W. H. Lai , H. Wang , L. Zheng , Q. Jiang , Z. C. Yan , L. Wang , H. Yoshikawa , D. Matsumura , Q. Sun , Y. X. Wang , Angew. Chem., Int. Ed. 2020, 132, 22355.

[67] H. Wang , W. Zhang , J. Xu , Z. Guo , Adv. Funct. Mater. 2018, 28, 1707520.

[68] D. Ma , Y. Li , J. Yang , H. Mi , S. Luo , L. Deng , C. Yan , M. Rauf , P. Zhang , X. Sun , Adv. Funct. Mater. 2018, 28, 1705537.

[69] Y. Shan , Y. Li , H. Pang , Adv. Funct. Mater. 2020, 30, 2001298.

[70] Z. Yan , Y. Liang , J. Xiao , W. Lai , W. Wang , Q. Xia , Y. Wang , Q. Gu , H. Lu , S. L. Chou , Adv. Mater. 2020, 32, 1906700. 10.1002/adma.201906700 31943381

[71] T. Yang , B. Guo , W. Du , M. K. Aslam , M. Tao , W. Zhong , Y. Chen , S. J. Bao , X. Zhang , M. Xu , Adv. Sci. 2019, 6, 1901557. 10.1002/advs.201901557 PMC6891912 31832316

[72] Y. Wang , Y. Lai , J. Chu , Z. Yan , Y. X. Wang , S. L. Chou , H. K. Liu , S. X. Dou , X. Ai , H. Yang , Adv. Mater. 2021, 33, 2100229. 10.1002/adma.202100229 33733506

[73] M. K. Aslam , I. D. Seymour , N. Katyal , S. Li , T. Yang , S.‐j. Bao , G. Henkelman , M. Xu , Nat. Commun. 2020, 11, 1. 33067473 10.1038/s41467-020-19078-0 PMC7568557

[74] H. Liu , W. Pei , W.‐H. Lai , Z. Yan , H. Yang , Y. Lei , Y.‐X. Wang , Q. Gu , S. Zhou , S. Chou , ACS Nano 2020, 14, 7259. 32433868 10.1021/acsnano.0c02488

[75] Z. Yan , Y. Liang , W. Hua , X.‐G. Zhang , W. Lai , Z. Hu , W. Wang , J. Peng , S. Indris , Y. Wang , ACS Nano 2020, 14, 10284. 32672932 10.1021/acsnano.0c03737

[76] A. Ghosh , A. Kumar , T. Das , A. Ghosh , S. Chakraborty , M. Kar , D. R. MacFarlane , S. Mitra , Adv. Funct. Mater. 2020, 30, 2005669.

[77] T. Wu , M. Jing , L. Yang , G. Zou , H. Hou , Y. Zhang , Y. Zhang , X. Cao , X. Ji , Adv. Energy Mater. 2019, 9, 1803478.

[78] J. Yan , W. Li , R. Wang , P. Feng , M. Jiang , J. Han , S. Cao , Z. Zhang , K. Wang , K. Jiang , ACS Energy Lett. 2020, 5, 1307.

[79] S. Zhang , T. P. Pollard , X. Feng , O. Borodin , K. Xu , Z. Li , ACS Energy Lett. 2020, 5, 1070.

